# Supplementary material for: Efficient experimental characterization of the GPCRome via deep receptor scanning
Source: Nat Commun. 2026 Aug 14;17:9796. doi: 10.1038/s41467-026-76564-7 (PMC13575135; doi:10.1038/s41467-026-76564-7)
Supplement: Supplementary file 1 [file 41467_2026_76564_MOESM1_ESM.pdf]

Supplementary Information for:

Efficient Experimental Characterization of the GPCRome via Deep Receptor Scanning

Austin Tedman,[1] Muskan Goel,[1] Sohan Shah,[1] Matthew K. Howard,[2,3] Laura M. Chamness,[4] Antonio Bonifasi,[1] Ismaila Adams,[5] Jacklyn M. Gallagher,[1] Wesley D. Penn,[6] Katarina Nemec,[7] Eli F. McDonald,[7] Brianna N. Corman,[8] J. Paul Robinson,[8] Carol Beth Post,[5] Patricia L. Clark,[4] M. Madan Babu,[7] Aashish Manglik,[3] Charles P. Kuntz,[1*] Willow Coyote-Maestas,[2*] and Jonathan P. Schlebach[1*]

[1]The James Tarpo Jr. and Margaret Tarpo Department of Chemistry, Purdue University, West Lafayette, IN, USA
[2]Department of Bioengineering and Therapeutic Science, University of California, San Francisco, San Francisco, CA, USA
[3]Department of Pharmaceutical Chemistry, University of California, San Francisco, San Francisco, CA, USA
[4]Department of Chemistry and Biochemistry, University of Notre Dame, Notre Dame, IN, USA
[5]The Borch Department of Medicinal Chemistry and Molecular Pharmacology, Purdue University, West Lafayette, IN, USA
[6]Department of Chemistry, Indiana University, Bloomington, IN, USA
[7]Center of Excellence for Data Driven Discovery, Department of Structural Biology, St. Jude Children's Research Hospital, Memphis, TN, USA
[8]Weldon School of Biomedical Engineering, Purdue University, West Lafayette, IN, USA

*Corresponding Authors: jschleba (at) purdue.edu, willow.coyote-maestas (at) ucsf.edu, and cpkuntz (at) purdue.edu

Contents:

-Supplementary Figure 1
-Supplementary Figure 2
-Supplementary Figure 3
-Supplementary Figure 4
-Supplementary Figure 5
-Supplementary Figure 6
-Supplementary Figure 7
-Supplementary Table 1
-Supplementary Table 2
-Supplementary Table 3

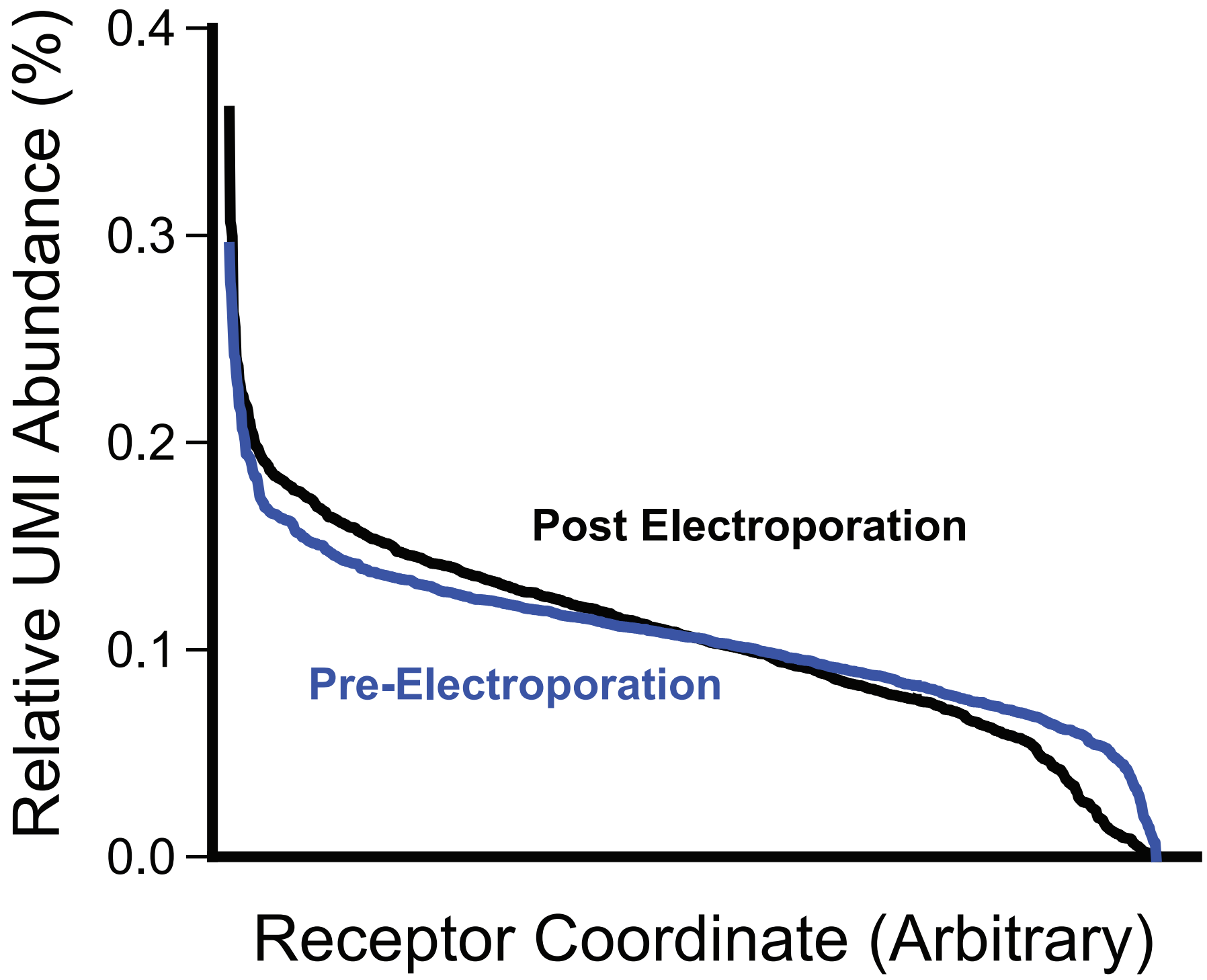

Supplementary Figure 1. Relative Abundance of GPCR Plasmids within Pooled Libraries. 946 individual plasmid preparations were hand-mixed at an equal stoichiometric ratio prior to electroporation of the pool into *E. coli*. The plasmid pool was then purified from *E. coli* prior to comparison of the relative abundance of each GPCR-associated UMI in the initial pool and the final plasmid preparation by deep sequencing. Individual UMIs are plotted onto an arbitrary coordinate according to the percentage of the total reads withn each plasmid population. Overall, the variance between individual UMIs is typically modest and is remains similar after transformation into *E. coli*.

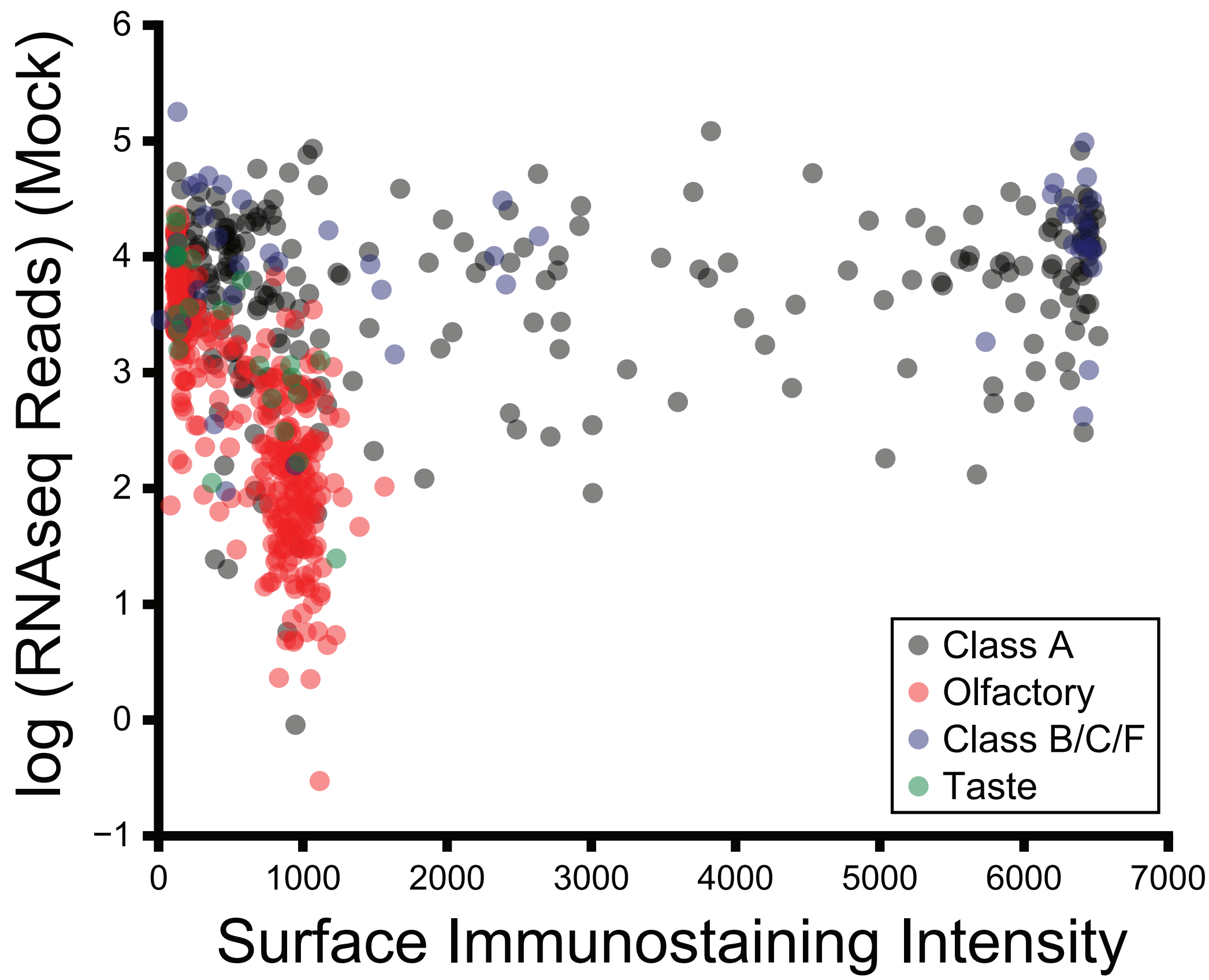

Supplementary Figure 2. GPCR Transcript Abundance and Surface Expression. A scatterplot depicts the $\log_{10}$ transformation of the number reads per kilobase for each GPCR-encoding transcript relative to the plasma membrane expression of the corresponding receptor protein. The number pf transcript reads for each receptor within a mock transfection sample were subtracted from the number of reads for the corresponding transcript within a pool of recombinant cells expressing GPCRs in order to correct for any endogenous receptor transcripts, which should not influence the immunostaining of the HA-tagged receptors. The RNAseq counts and surface immunostaining intensities were gathered in biological triplicate (n = 3 for both data sets).Transcripts are colored according to the class of the receptor they encode, for reference

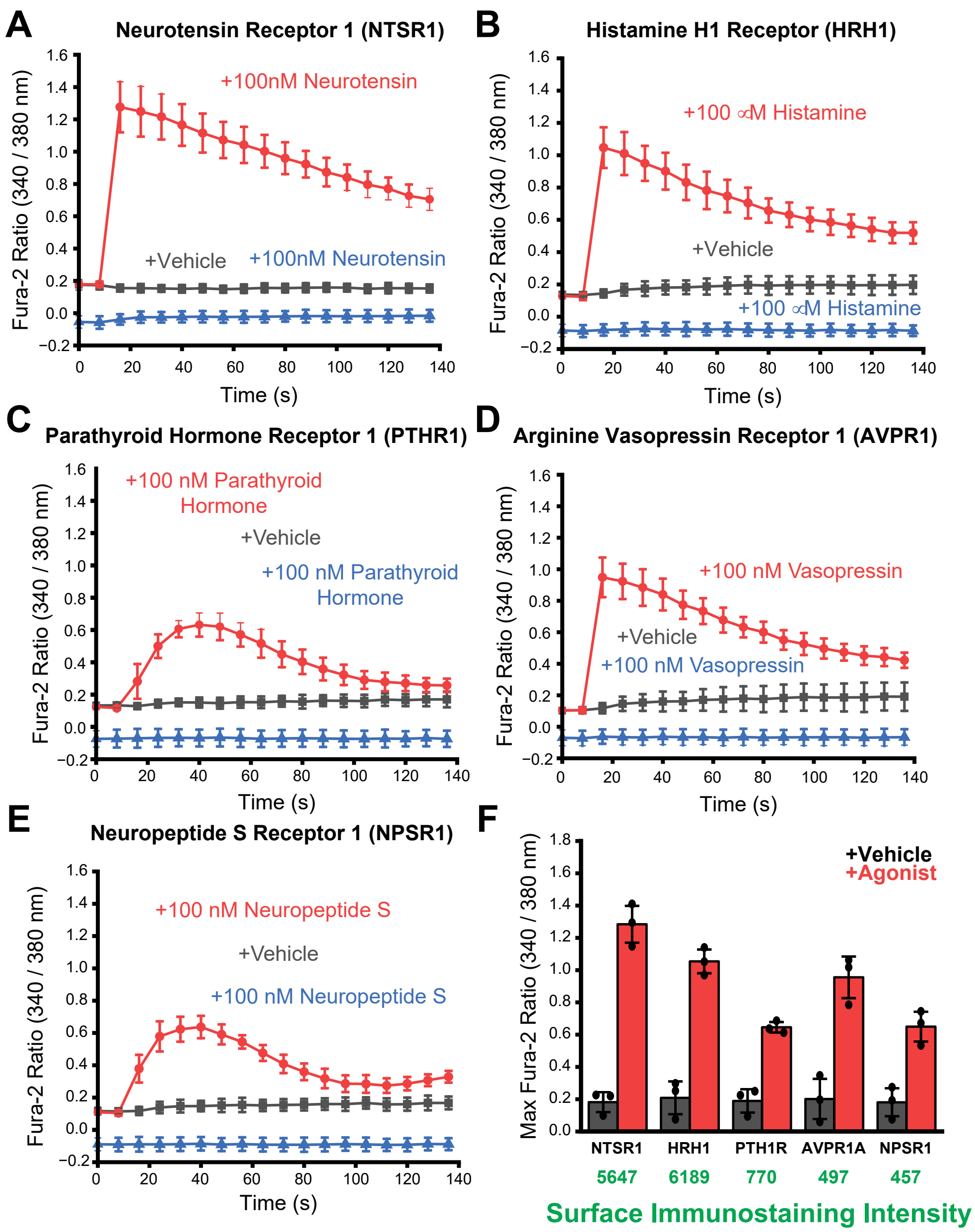

Supplementary Figure 3. Signaling Activity of GPCRs with Divergent Plasma Membrane Expression. The signaling activity of five GPCRs with varying plasma membrane expression was compared in the context of recombinant HEK293T cells by monitoring calcium fluxes generated in the presence of agonists. A series of recombinant HEK293T cell lines expressing A) NTSR1, B) HRH1, C) PTHR1, D) AVPR1, or E) NPSR1 were incubated with a calcium-sensitive Fura-2 dye prior to introduction of agonists. A series of line plots depict the change in the Fura-2 fluorescence emis-sion ratio over time after the addition of agonist (red) or vehicle (gray) to GPCR-expressing cells or addition of agonist to mock transfected cells (blue). F) A bar plot depicts the peak emission ratio achieved for cells expressing each receptor under each condition. The corresponding surface immunostaining intensities of each receptor by deep receptor scanning is shown in green for reference. Emission ratio values reflect the average of nine technical replicates collected across three biological replicates and error bars reflect the standard error. The maximum values for each given receptor for each replicate for each condition are indicated. We note that the transcripts of these receptors were not observed within RNA-Seq measurements of the parental cell line. Together, these results demonstrate the the plasma membrane expression of recombinantly expressed GPCRs does not limit signal transduction in the context of this cell line.

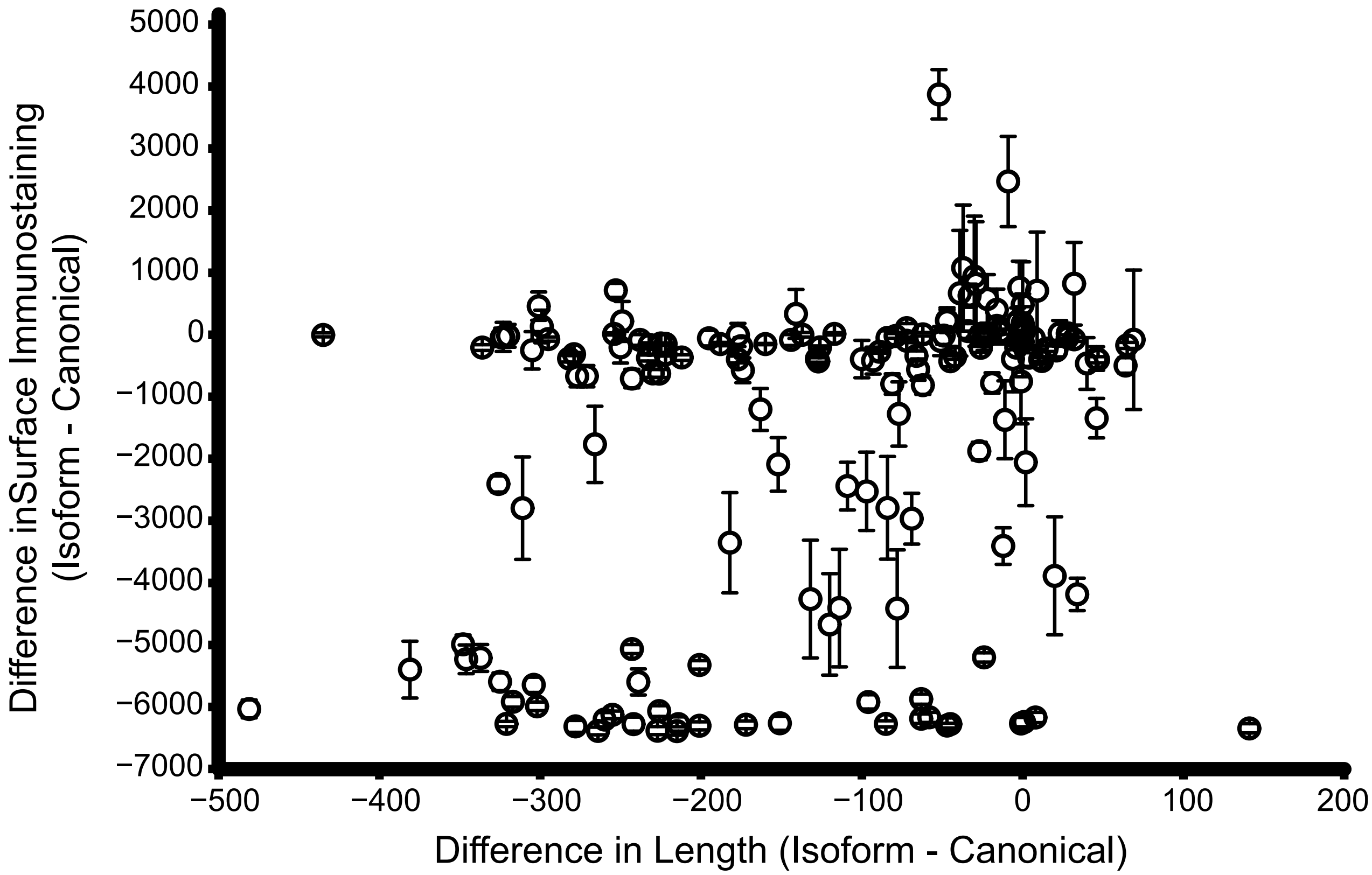

Supplementary Figure 4. Trends in the Plasma Membrane Expression of GPCR isoforms with Varying Length. A scatterplot depicts the change in the plasma membrane expression of each isoform in relation to its corresponding change in protein length according to deep receptor scanning measurements across three biological replicates for each GPCR isoform (n = 3). Bars depicting the standard deviation of the difference betweenan isoform and its canonical form are shown. Changes in expression and length are cast in terms of the isoform receptor value minus the canonical receptor value.

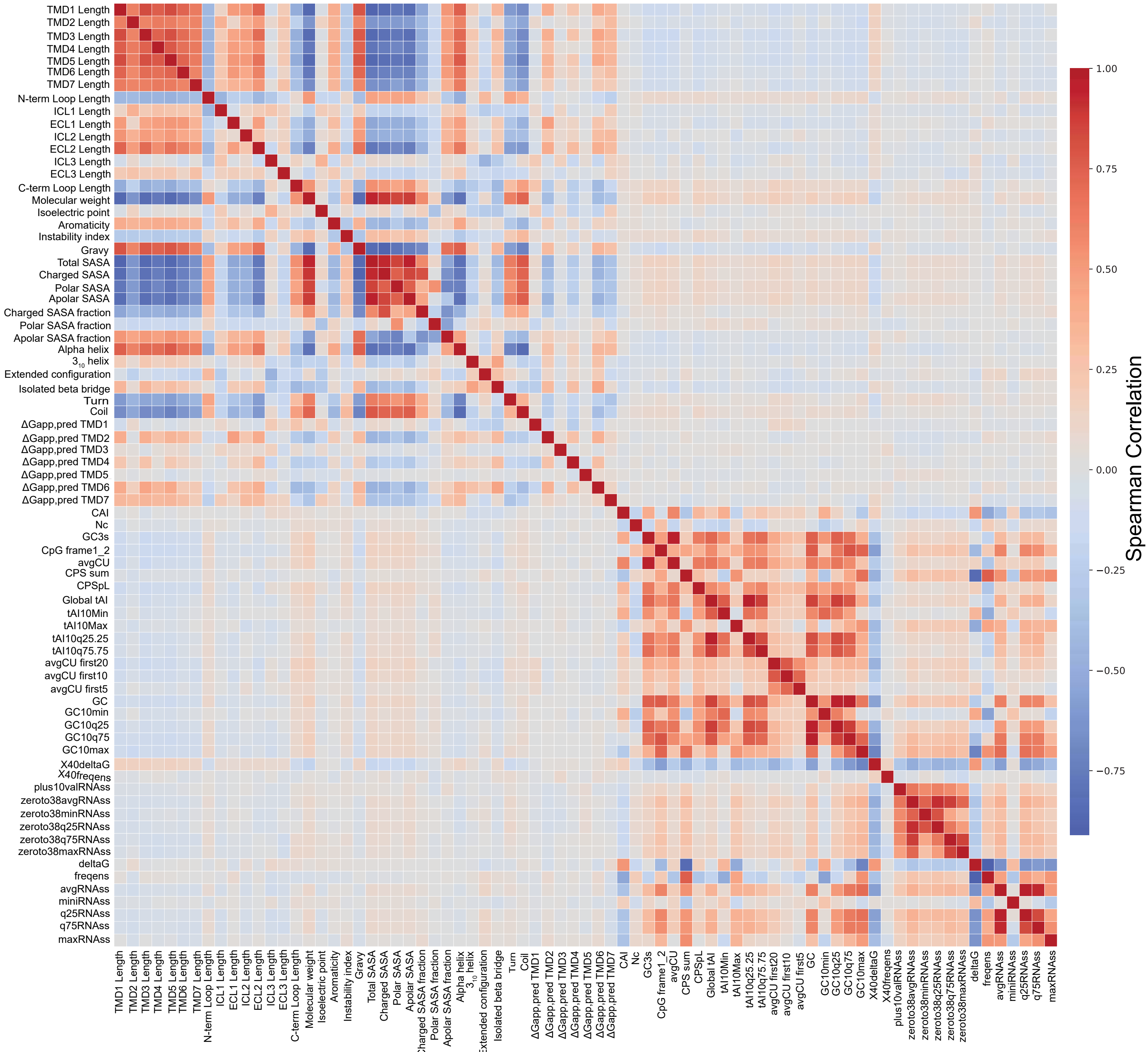

Supplementary Figure 5. Correlation Heatmap showing pairwise assocation among all features. A heat map depicts the magnitude of the Spearman correlation coefficients for each pair of machine learning features. Each cell represents the monotonic association between a pair of features, with values ranging from +1 (strong positive correlation) to -1 (strong negative correlation).

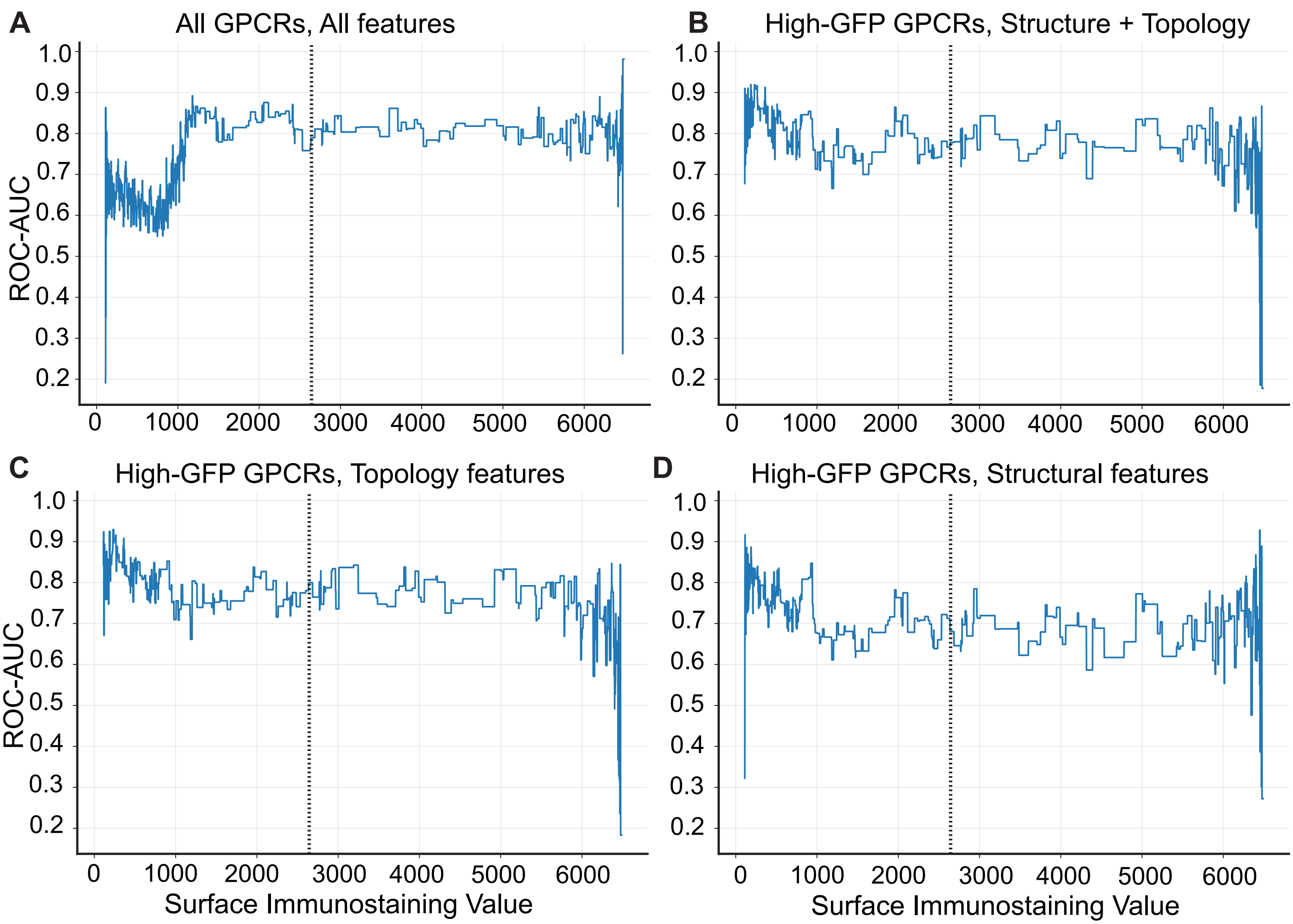

Supplementary Figure 6. Impact of Cutoff Values on the Performance of Random Forest Classier Models. A series of random forest models with varying PME cutoff values were trained to classify the plasma membrane expression (PME) of A) All GPCRs or B-D) High-GFP GPCRs based on A) all features, B) structural and topological features, C) topological features only, or D) structural features only. Each cutoff value was used to classify GPCRs as either low or high PME, and ROC-AUC was calculated for each model trained with each cutoff value. The ROC-AUC for each model is plotted against its corresponding PME cutoff value. The black dotted vertical line indicates the position of the PME cutoff of 2644, which was used for the models shown in Figure 6. Cutoffs were skipped when only one class was present or when either class contained fewer than 5 receptors.

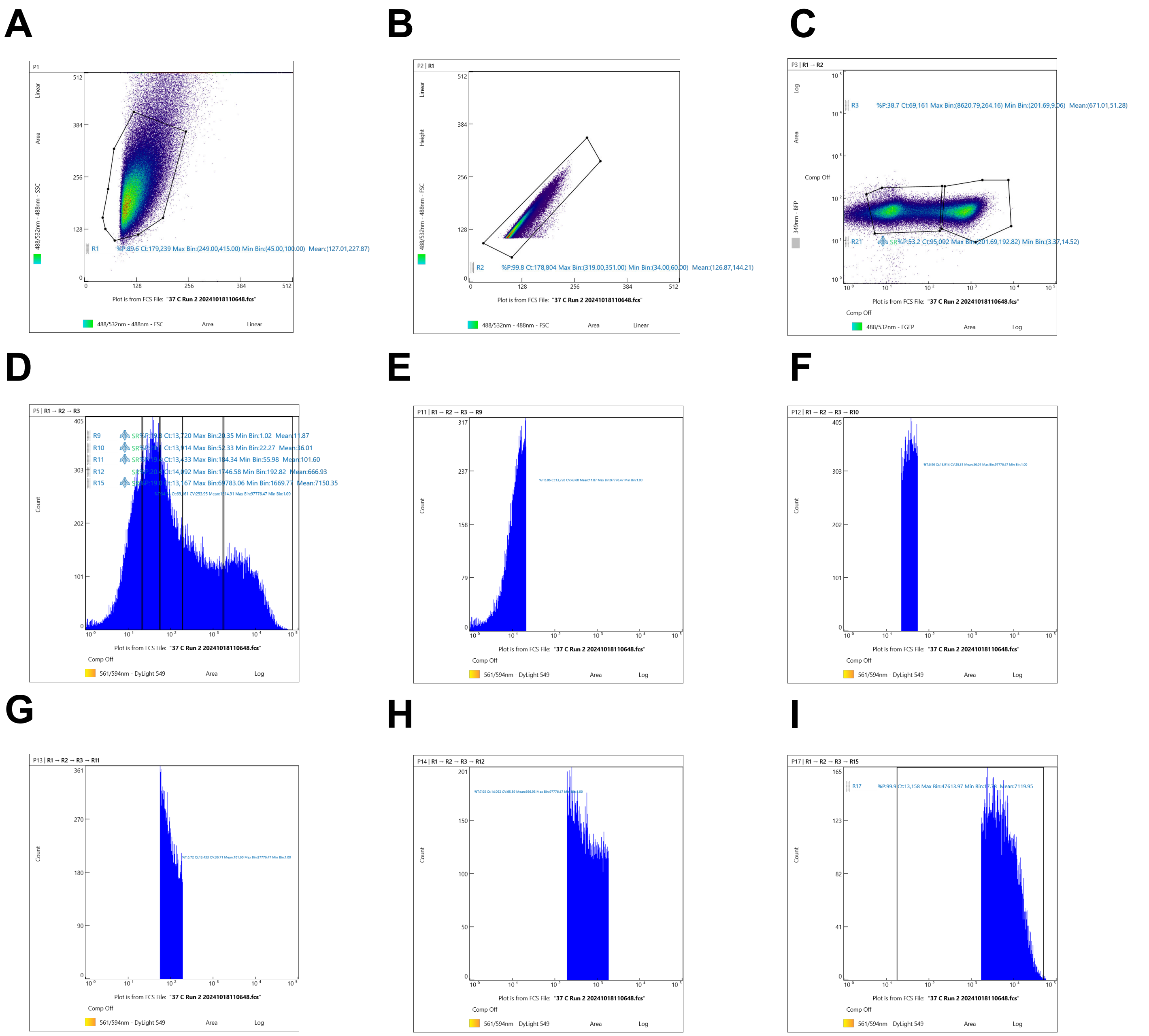

Supplementary Figure 7. Gating Strategies for Deep Receptor Scanning. A representative depiction of oneof the three biological replicates sorting strategy depicting how events are isolated into A) cells, B) singlets before C) being fractionated based on GFP levels. The GFP+ population of cells (right gate in C)) were then D) further fractionated into 5 equal quintiles based on their relative surface immunostaining. E) - I) are individual windows of each bin to record the mean florescent intensity of each gate. Reads gathered from C) were used to calculate the relative GFP levels of Figure 2 and Figure 5A, while the reads gathered from E) - I) were used to calculate the surface immunostaining levels featured in Figure 4, Figure 5B and Figure 6.

Supplementary Table 1. Supervised Model Performance

Performance metrics for the top four models according to AUROC in each category are shown.

Models trained to classify the full set of GPCRs (n= 780) w/ the full feature set (n= 75).*

Baseline model	Balanced Accuracy	F1-Score	Precision	Recall	ROC-AUC
Random Forest**	0.643	0.422	0.489	0.381	0.818
**Comparison model**	**Balanced Accuracy**	**F1-Score**	**Precision**	**Recall**	**ROC-AUC**
SVM Linear	0.728	0.491	0.352	0.815	0.776
SVM RBF	0.714	0.487	0.368	0.719	0.785
Logistic Regression	0.717	0.490	0.374	0.718	0.772

Models trained to classify GPCRs with stable transcripts (n= 452) w/ topological and structural features (n= 40).*

Baseline model	Balanced Accuracy	F1-Score	Precision	Recall	ROC-AUC
Random Forest**	0.658	0.524	0.604	0.470	0.752
**Comparison model**	**Balanced Accuracy**	**F1-Score**	**Precision**	**Recall**	**ROC-AUC**
SVM Linear	0.569	0.488	0.374	0.724	0.607
Logistic Regression	0.620	0.518	0.452	0.613	0.692
Naïve Bayes	0.624	0.505	0.492	0.521	0.697

Models trained to classify GPCRs with stable transcripts (n= 452) w/ topological features (n= 27).*

Baseline model	Balanced Accuracy	F1-Score	Precision	Recall	ROC-AUC
Random Forest**	0.644	0.504	0.579	0.457	0.744
**Comparison model**	**Balanced Accuracy**	**F1-Score**	**Precision**	**Recall**	**ROC-AUC**
SVM Linear	0.638	0.569	0.414	0.908	0.632
Logistic Regression	0.625	0.527	0.456	0.630	0.689
Neural Network	0.500	0.299	0.199	0.600	0.539

Models trained to classify GPCRs with stable transcripts (n= 452) w/ structural features (n= 13).*

Baseline model	Balanced Accuracy	F1-Score	Precision	Recall	ROC-AUC
Random Forest**	0.593	0.441	0.470	0.427	0.688
**Comparison model**	**Balanced Accuracy**	**F1-Score**	**Precision**	**Recall**	**ROC-AUC**
SVM RBF	0.611	0.530	0.416	0.731	0.619
SVM Linear	0.516	0.426	0.347	0.572	0.529
Logistic Regression	0.570	0.450	0.403	0.513	0.582

**Certain isoforms lack seven transmembrane domains and do not contain a full set of topological features. For this reason, they were excluded from model training. GPCRs we considered to be encoded by stable transcripts if recombinant cells expressing these cells were not statistically enriched within the sub-population of low-GFP cells.*

**Models shaded in gray correspond to those featured in Figure 6.

Supplementary Table 2. Relative Contribution of Features in Different Models

Rank	All GPCRs All Features		High-GFP GPCRs Structure + Topology		High-GFP GPCRs Topology Features		High-GFP GPCRs Structural Features	
	Feature	Feature Importance	Feature	Feature Importance	Feature	Feature Importance	Feature	Feature Importance
1	TMD1 Length	0.03986	TMD1 Length	0.0261198	TMD1 Length	0.047766	Total SASA	0.10416320
2	TMD2 Length	0.011294	TMD2 Length	0.0201237	TMD2 Length	0.02962769	Charged SASA	0.089916
3	TMD3 Length	0.042552	TMD3 Length	0.0240073	TMD3 Length	0.03872244	sasa plr	0.068497
4	TMD4 Length	0.012476	TMD4 Length	0.0177220	TMD4 Length	0.02759578	sasa aplr	0.080524
5	TMD5 Length	0.025854	TMD5 Length	0.0208994	TMD5 Length	0.030627	f_crg	0.064925
6	TMD6 Length	0.011189	TMD6 Length	0.0174769	TMD6 Length	0.02775532	f_plr	0.058854
7	TMD7 Length	0.010330	TMD7 Length	0.0164976	TMD7 Length	0.02472043	f_aplr	0.057252
8	N-term Loop Length	0.013730	N-term Loop Length	0.032529	N-term Loop Length	0.04396154	Alpha Helix	0.099997
9	ICL1 Length	0.008349	ICL1 Length	0.0194606	ICL1 Length	0.027935	3_{10} Helix Content	0.082937
10	ECL1 Length	0.011758	ECL1 Length	0.023272	ECL1 Length	0.034732	extended_configuration	0.05468029
11	ICL2 Length	0.010362	ICL2 Length	0.0200846	ICL2 Length	0.032398	isolated beta bridge	0.065759
12	ECL2 Length	0.040180	ECL2 Length	0.0291135	ECL2 Length	0.043461	turn	0.079411
13	ICL3 Length	0.007087	ICL3 Length	0.0168760	ICL3 Length	0.02599839	coil	0.093085
14	ECL3 Length	0.013684	ECL3 Length	0.0252995	ECL3 Length	0.033203		
15	C-term Loop Length	0.023710	C-term Loop Length	0.0295878	C-term Loop Length	0.042994		
16	Molecular weight	0.039441	Molecular weight	0.033936	Molecular weight	0.051168		
17	Isoelectric_Point	0.009251	Isoelectric_Point	0.0205114	Isoelectric_Point	0.031539		
18	Aromaticity	0.012504	Aromaticity	0.022481	Aromaticity	0.033639		
19	instability_index	0.010491	instability_index	0.0243622	instability_index	0.03590598		
20	gravy	0.021469	gravy	0.0214195	gravy	0.034897		
21	ΔGapp,pred TMD1	0.009849	ΔGapp,pred TMD1	0.0248540	ΔGapp,pred TMD1	0.036292		
22	ΔGapp,pred TMD2	0.017400	ΔGapp,pred TMD2	0.030739	ΔGapp,pred TMD2	0.04545		
23	ΔGapp,pred TMD3	0.009715	ΔGapp,pred TMD3	0.019609	ΔGapp,pred TMD3	0.02952387		
24	ΔGapp,pred TMD4	0.012979	ΔGapp,pred TMD4	0.023471	ΔGapp,pred TMD4	0.037086		
25	ΔGapp,pred TMD5	0.009569	ΔGapp,pred TMD5	0.0239928	ΔGapp,pred TMD5	0.03246884		
26	ΔGapp,pred TMD6	0.032793	ΔGapp,pred TMD6	0.071222	ΔGapp,pred TMD6	0.0927		
27	ΔGapp,pred TMD7	0.009779	ΔGapp,pred TMD7	0.0187724	ΔGapp,pred TMD7	0.027834		
28	Total SASA	0.048656	Total SASA	0.032621				
29	Charged SASA	0.032946	Charged SASA	0.027229				
30	sasa plr	0.011109	sasa plr	0.0188665				

31	sasa aplr	0.037735	sasa aplr	0.026807				
32	f_crg	0.010488	f_crg	0.021135				
33	f_plr	0.008899	f_plr	0.0182271				
34	f_aplr	0.007259	f_aplr	0.0178868				
35	Alpha Helix	0.035475	Alpha Helix	0.0319988				
36	3_{10} Helix Content	0.012183	3_{10} Helix Content	0.034067				
37	extended_configuration	0.009296	extended_configuration	0.0158215				
38	isolated beta bridge	0.025631	isolated beta bridge	0.0213079				
39	turn	0.016868	turn	0.0270167				
40	coil	0.036439	coil	0.032574				
41	CAI	0.005704						
42	Nc	0.007185						
43	GC3s	0.005024						
44	CpG_frame1_2	0.005434						
45	avgCU	0.00539						
46	CPS_sum	0.0061						
47	CPSpL	0.005795						
48	Global_tAI	0.006516						
49	tAI10Min	0.006979						
50	tAI10Max	0.007019						
51	tAI10q25.25.	0.006778						
52	tAI10q75.75.	0.007824						
53	avgCU_first20	0.008274						
54	avgCU_first10	0.007477						
55	avgCU_first5	0.008004						
56	GC	0.005902						
57	GC10min	0.003882						
58	GC10q25	0.00274						
59	GC10q75	0.002623						
60	GC10max	0.003129						
61	X40deltaG	0.008136						
62	X40freqens	0.009005						
63	plus10valRNAss	0.007036						
64	zeroto38avgRNAss	0.007297						
65	zeroto38minRNAss	0.008987						

66	zeroto38q25RNAss	0.008321						
67	zeroto38q75RNAss	0.00637						
68	zeroto38maxRNAss	0.006988						
69	deltaG	0.006505						
70	freqens	0.006236						
71	avgRNAss	0.00644						
72	minRNAss	0.009164						
73	q25RNAss	0.007022						
74	q75RNAss	0.006246						
75	maxRNAss	0.007819						

Supplementary Table 3. Overview of Machine Learning Features

Transcript Feature Name	Description
CAI	Codon Adaptation Index. Category: Overall Codon Usage. Calculation Method/Tool: codonW 1.4.4
Nc	Effective number of codons. Category: Overall Codon Usage. Calculation Method/Tool: codonW 1.4.4
GC3s	GC content at the synonymous position. Category: Nucleotide. Calculation Method/Tool: Custom
CpG_frame1_2	Frequency of CG di-nucleotides. Category: Nucleotide. Calculation Method/Tool: Custom
avgCU	Average Codon Usage. Category: 5' Codon Usage. Calculation Method/Tool: Biopython
CPS_sum	Sum of Codon Pair Score values. Category: Codon Pair Score. Calculation Method/Tool: Coleman et al., 2008
CPSpL	Codon Pair Bias. Category: Codon Pair Score. Calculation Method/Tool: Coleman et al., 2008
Global_tAI	tRNA Adaptation Index. Category: tRNA Adaptation Index. Calculation Method/Tool: codonR
tAI10Min	Minimum tAI score over 10 codon windows. Category: tRNA Adaptation Index. Calculation Method/Tool: codonR
tAI10Max	Maximum tAI score over 10 codon windows. Category: tRNA Adaptation Index. Calculation Method/Tool: codonR
TAI10q25.25.	25th percentile of tAI scores over 10 codon windows. Category: tRNA Adaptation Index. Calculation Method/Tool: codonR
TAI10q75.75.	75th percentile of tAI scores over 10 codon windows. Category: tRNA Adaptation Index. Calculation Method/Tool: codonR
avgCU_first20	Codon Usage over the first 20 codons. Category: 5' Codon Usage. Calculation Method/Tool: Biopython
avgCU_first5	Codon Usage over the first 5 codons. Category: 5' Codon Usage. Calculation Method/Tool: Biopython
avgCU_first10	Codon Usage over the first 10 codons. Category: 5' Codon Usage. Calculation Method/Tool: Biopython
GC	Overall GC content. Category: Nucleotide. Calculation Method/Tool: Custom
GC10min	Minimum %GC over 10 codon windows. Category: Nucleotide. Calculation Method/Tool: Custom
GC10q25	25th percentile of %GC over 10 codon windows. Category: Nucleotide. Calculation Method/Tool: Custom
GC10q75	75th percentile of %GC over 10 codon windows. Category: Nucleotide. Calculation Method/Tool: Custom
GC10max	Maximum %GC over 10 codon windows. Category: Nucleotide. Calculation Method/Tool: Custom
X40deltaG	ŒîG of the lowest free energy structure for the first 40 codons. Category: 5' RNA Structure. Calculation Method/Tool: RNAfold
X40freqens	Frequency of the lowest free energy structure within the ensemble for the first 40 codons. Category: 5' RNA Structure. Calculation Method/Tool: RNAfold
plus10valRNAss	Average hybridization probability centered around +10 base, i.e. average of +5 to +15. Category: 5' RNA Structure. Calculation Method/Tool: RNAfold
zeroto38avgRNAss	Average hybridization probability over 10 base windows from 0 to +38. Category: 5' RNA Structure. Calculation Method/Tool: NUPACK

zeroto38minRNAss	Minimum hybridization probability over 10 base windows from 0 to +38. Category: 5' RNA Structure. Calculation Method/Tool: NUPACK
zeroto38q25RNAss	25th percentile of hybridization probability over 10 base windows from 0 to +38. Category: 5' RNA Structure. Calculation Method/Tool: NUPACK
zeroto38q75RNAss	75th percentile of hybridization probability over 10 base windows from 0 to +38. Category: 5' RNA Structure. Calculation Method/Tool: NUPACK
zeroto38maxRNAss	Maximum hybridization probability over 10 base windows from 0 to +38. Category: 5' RNA Structure. Calculation Method/Tool: NUPACK
deltaG	ŒîG of the lowest free energy structure. Category: Overall RNA Structure. Calculation Method/Tool: RNAfold
freqens	Frequency of the lowest free energy structure within the ensemble. Category: Overall RNA Structure. Calculation Method/Tool: RNAfold
avgRNAss	Average hybridization probability over 10 base windows. Category: Overall RNA Structure. Calculation Method/Tool: NUPACK
minRNAss	Minimum hybridization probability over 10 base windows. Category: Overall RNA Structure. Calculation Method/Tool: NUPACK
q25RNAss	25th percentile of hybridization probability over 10 base windows. Category: Overall RNA Structure. Calculation Method/Tool: NUPACK
q75RNAss	75th percentile of hybridization probability over 10 base windows. Category: Overall RNA Structure. Calculation Method/Tool: NUPACK
maxRNAss	Maximum hybridization probability over 10 base windows. Category: Overall RNA Structure. Calculation Method/Tool: NUPACK

**Protein Feature name**	**Description**
Length_without_SP	Length of the protein after removal of the signal peptide.
TMD1-7 Length	Normalized lengths of each transmembrane domain (TMD1–TMD7), relative to the full-length protein. TMDs are predicted using **DeepTMHMM**, and their coordinates are refined using the **von Heijne transfer free energy scale** von_heijne_scan_functions_v39_signal_pep_topcons.py (https://github.com/schebachlab/PRF-Search). The lengths are then normalized to the length of protein
N-term Loop Length	Normalized length of the N-terminal extracellular region preceding TMD1.
ICL1-3 Length	Normalized lengths of the intracellular loops immediately following TMD1, TMD2, and TMD3, respectively.
ECL1-3 Length	Normalized lengths of the extracellular loops immediately preceding TMD2, TMD3, and TMD4, respectively.
C-term Loop Length	Normalized length of the C-terminal intracellular region following TMD7.
$\Delta G_{app,pred}$ TMD1-7	Free energy of membrane insertion (ΔGAPP,PRED) for each TMD (TMD1–TMD7), computed using the **von Heijne transfer free energy scale** via von_heijne_scan_functions_v39_signal_pep_topcons.py (https://github.com/schebachlab/PRF-Search). These ΔGAPP,PRED values reflect the biophysical favorability of each TMD's insertion into the membrane.
Molecular_Weight	The molecular weight of the protein (in Daltons), computed from its amino acid composition.
Isoelectric_Point	The theoretical pH at which the protein carries no net charge.
Aromaticity	The fraction of aromatic amino acids (Phe, Trp, Tyr) present in the protein.
instability_index	Predicts the in vitro stability of a protein based on dipeptide composition. Values greater than 40 suggest stability; values less 40 indicate instability. Defined by Guruprasad et al. (1990):(https://pubmed.ncbi.nlm.nih.gov/2075190/)
gravy	Average hydrophobicity of the amino acids in the sequence using the Kyte–Doolittle scale: https://doi.org/10.1016/0022-2836(82)90515-0.

Total SASA**	The total solvent-accessible surface area (SASA) of the protein, calculated using FreeSASA:<br>https://freesasa.github.io/.
sasa_crg**<br>sasa_plr**<br>sasa_aplr**	Contributions to total SASA from:<br>– Charged SASA: Charged residues (Asp, Glu, Lys, Arg,His).<br>– SASA plr: Polar residues (Ser, Thr, Asn, Gln, Tyr, Cys).<br>– SASA aplr: Apolar (non-polar) residues (Ala, Val, Ile, Leu, Met, Phe, Trp, Pro,<br>Gly).
f_crg**<br>f_plr**<br>f_aplr**	Fractions of the total SASA contributed by charged, polar, and apolar residues, respectively<br>(range: 0–1).
alpha_helix**	Fraction of residues in alpha-helical conformation, as assigned by STRIDE.
3_{10} Helix Content**	Fraction of residues in 3_{10} helix conformation.
extended_configuration**	Fraction of residues in extended strand (beta-sheet-like) conformations.
isolated_beta_bridge**	Fraction of residues forming isolated beta-bridges (not part of extended beta-sheets).
Turn**	Fraction of residues in turns—tight loops reversing the direction of the peptide backbone.
Coil**	Fraction of residues in unordered, coil-like conformations.

*** Columns shaded in gray represent protein features derived from AF2 structures, while the remaining columns correspond to sequence-level protein or transcript features.*
