# Supplementary material for: Efficient experimental characterization of the GPCRome via deep receptor scanning
Source: Nat Commun. 2026 Aug 14;17:9796. doi: 10.1038/s41467-026-76564-7 (PMC13575135; doi:10.1038/s41467-026-76564-7)
Supplement: Supplementary file 2 [file 41467_2026_76564_MOESM2_ESM.pdf]

Description of Additional Supplementary Files

File name: Supplementary Data 1
Description: Descriptive measurements of GPCR biosynthesis. This file contains a table of measurements for the various parameters outlined in the manuscript, including but not limited to surface immunostaining, RNAseq and Riboseq reads for canonical receptors and the isoforms outlined in this manuscript.

File name: Supplementary Data 2
Description: Machine Learning Features. This file contains a table of the parameters used for the machine learning performed in this manuscript for all receptors.

File name: Supplementary Data 3
Description: Plasmid Sequences. This file contains a list of the exact sequences for all the plasmids used in this study.
