# Supplementary material for: Efficient experimental characterization of the GPCRome via deep receptor scanning
Source: Nat Commun. 2026 Aug 14;17:9796. doi: 10.1038/s41467-026-76564-7 (PMC13575135; doi:10.1038/s41467-026-76564-7)
Supplement: Supplementary file 3 [file 41467_2026_76564_MOESM6_ESM.pdf]

nature portfolio

Corresponding author(s): Jonathan Schlebach, Charles Kuntz, and Willow Coyote-Maestas

Last updated by author(s): Jul 22, 2026

Reporting Summary

Nature Portfolio wishes to improve the reproducibility of the work that we publish. This form provides structure for consistency and transparency in reporting. For further information on Nature Portfolio policies, see our Editorial Policies and the Editorial Policy Checklist.

Statistics

For all statistical analyses, confirm that the following items are present in the figure legend, table legend, main text, or Methods section.

n/a	Confirmed	
☐	☒	The exact sample size (*n*) for each experimental group/condition, given as a discrete number and unit of measurement
☐	☒	A statement on whether measurements were taken from distinct samples or whether the same sample was measured repeatedly
☐	☒	The statistical test(s) used AND whether they are one- or two-sided<br>*Only common tests should be described solely by name; describe more complex techniques in the Methods section.*
☐	☒	A description of all covariates tested
☒	☐	A description of any assumptions or corrections, such as tests of normality and adjustment for multiple comparisons
☐	☒	A full description of the statistical parameters including central tendency (e.g. means) or other basic estimates (e.g. regression coefficient) AND variation (e.g. standard deviation) or associated estimates of uncertainty (e.g. confidence intervals)
☐	☒	For null hypothesis testing, the test statistic (e.g. F, t, r) with confidence intervals, effect sizes, degrees of freedom and P value noted<br>*Give P values as exact values whenever suitable.*
☒	☐	For Bayesian analysis, information on the choice of priors and Markov chain Monte Carlo settings
☒	☐	For hierarchical and complex designs, identification of the appropriate level for tests and full reporting of outcomes
☒	☐	Estimates of effect sizes (e.g. Cohen's *d*, Pearson's *r*), indicating how they were calculated

Our web collection on statistics for biologists contains articles on many of the points above.

Software and code

Policy information about availability of computer code

Data collection	Ribo-Sequencing data were trimmed with cutadapt and UMI-tools to remove Illumina linker regions and UMI sequences from each read, respectively. Non-coding RNA sequences were obtained from RNAcentral and removed from sequencing data using Bowtie2.
Data analysis	Deep Receptor Scanning calculations were carried out using a custom software package used previously (https://github.com/schlebachlab/DRS). Synonymous codon usage in HA-tagged GPCR transcript sequences were evaluated using the %MinMax algorithm. Codon usage data for H. sapiens was sourced from the HIVE-CUT database. The Riboseq and RNAseq reads were then mapped to GPCR transcript sequences using STAR. A meta-analysis of the ribosome pileups across different receptor sub-types was carried out using a custom software package (doi: 10.5281/zenodo.21081354, https://github.com/schlebachlab/DRS).

For manuscripts utilizing custom algorithms or software that are central to the research but not yet described in published literature, software must be made available to editors and reviewers. We strongly encourage code deposition in a community repository (e.g. GitHub). See the Nature Portfolio guidelines for submitting code & software for further information.

Data

Policy information about availability of data

All manuscripts must include a data availability statement. This statement should provide the following information, where applicable:

- Accession codes, unique identifiers, or web links for publicly available datasets
- A description of any restrictions on data availability
- For clinical datasets or third party data, please ensure that the statement adheres to our policy

The pooled GPCR expression vector libraries described herein are available through Addgene (ID numbers 243681 and 243682). Individual GPCR expression plasmids will be freely shared with the research community upon request. HEK293T cells that were used to generate recombinant cell lines were a kind gift from Douglas Fowler. Sequencing data for deep receptor scanning have been deposited into the NCBI Database (Accession code: PRJNA1280020 [https://www.ncbi.nlm.nih.gov/bioproject/?term=PRJNA1280020]). Sequencing data for RNA-Seq experiments can be accessed through the Gene Expression Omnibus (Accession Code: GSE304607 [https://www.ncbi.nlm.nih.gov/geo/query/acc.cgi?acc=GSE304607]). Sequencing data for Ribo-Seq experiments can be accessed through the Gene Expression Omnibus (Accession Code: GSE307296 [https://www.ncbi.nlm.nih.gov/geo/query/acc.cgi?acc=GSE307296]). Processed data, including surface immunostaining intensity values, processed RNA-Seq read counts, Ribo-Seq footprint counts, and fold-enrichment values for each receptor can be found in Supplementary Data 1. Plasmid maps, structural models and all other raw experimental data have been deposited in a freely accessible Mendeley data directory (DOI:10.17632/3b4n36z4bg.3 [https://doi.org/10.17632/3b4n36z4bg.3]).

Research involving human participants, their data, or biological material

Policy information about studies with human participants or human data. See also policy information about sex, gender (identity/presentation), and sexual orientation and race, ethnicity and racism.

Reporting on sex and gender	N/A
Reporting on race, ethnicity, or other socially relevant groupings	N/A
Population characteristics	N/A
Recruitment	N/A
Ethics oversight	N/A

Note that full information on the approval of the study protocol must also be provided in the manuscript.

Field-specific reporting

Please select the one below that is the best fit for your research. If you are not sure, read the appropriate sections before making your selection.

☒ Life sciences ☐ Behavioural & social sciences ☐ Ecological, evolutionary & environmental sciences

For a reference copy of the document with all sections, see nature.com/documents/nr-reporting-summary-flat.pdf

Life sciences study design

All studies must disclose on these points even when the disclosure is negative.

Sample size	A sample size of three for the DRS scanning and RNAseq was determined to ensure robust reproducibility and statistical power. This sample size is the standard for the field. A sample size of one was used for the Ribo-seq experiment to identify general trends. No statistical tests were used, as the results are qualitative in nature.
Data exclusions	Enrichment scores were only calculated for receptors that were sampled at a frequency of at least 0.0005% within each subpopulation (i.e. 10 reads for low GFP, 50 reads for high GFP) across all 3 replicates. This was done to ensure that the reads ascribed to each GPCR was not merely sequencing noise.
Replication	Biological replicates consisting of 3 distinct cellular pools of the stably recombined HEK 293T cells were harvested on different days and sorted. All replication attempts were successful.
Randomization	Randomization is not relevant for these experiments.
Blinding	Blinding is not relevant for these experiments.

Reporting for specific materials, systems and methods

We require information from authors about some types of materials, experimental systems and methods used in many studies. Here, indicate whether each material, system or method listed is relevant to your study. If you are not sure if a list item applies to your research, read the appropriate section before selecting a response.

Materials & experimental systems

n/a	Involved in the study	
☐	☒	Antibodies
☐	☒	Eukaryotic cell lines
☒	☐	Palaeontology and archaeology
☒	☐	Animals and other organisms
☒	☐	Clinical data
☒	☐	Dual use research of concern
☒	☐	Plants

Methods

n/a	Involved in the study	
☒	☐	ChIP-seq
☐	☒	Flow cytometry
☒	☐	MRI-based neuroimaging

Antibodies

Antibodies used	Used a mouse monoclonal anti-Hemaglutinin antibody conjugated with Dylight 550 (2-2.2.14) supplied by Invitrogen. (Cat# 26183-D550) (RRID: AB_2533052)
Validation	This antibody was verified by the manufacturer using relative expression as a readout to validate that the antibody binds to the antigen.

Eukaryotic cell lines

Policy information about cell lines and Sex and Gender in Research

Cell line source(s)	The modified HEK293T were previously transduced with a lentiviral vector to contain a genomic "landing pad". The cells used in the study were donated by Douglas Fowler
Authentication	Modified HEK293T cells, which were a generous gift from Douglas Fowler (University of Washington), were authenticated using an STR profiling service offered by American Type Culture Collection (ATCC).
Mycoplasma contamination	Cells tested negative for mycoplasma using qPCR as verification.
Commonly misidentified lines (See ICLAC register)	We did not use any of these cell lines.

Plants

Seed stocks	N/A
Novel plant genotypes	N/A
Authentication	N/A

Flow Cytometry

Plots

Confirm that:

☒ The axis labels state the marker and fluorochrome used (e.g. CD4-FITC).

☒ The axis scales are clearly visible. Include numbers along axes only for bottom left plot of group (a 'group' is an analysis of identical markers).

☐ All plots are contour plots with outliers or pseudocolor plots.

☒ A numerical value for number of cells or percentage (with statistics) is provided.

Methodology

Sample preparation	The adherent cells were washed with phosphate buffered saline and harvested with 0.25% Trypsin-EDTA. HA-tagged GPCRs

Sample preparation	expressed at the plasma membrane were immunostained by incubating the cells with a DyLight550–conjugated anti-HA antibody for 30 minutes. Cells were then washed twice with phosphate buffered saline and passed through a 40 µm cell strainer to remove cellular aggregates prior to separation.
Instrument	A BIGFOOT 9 LASER 60P CELL SORTER (Model #: PL00285) was used to collect data and sort cells.
Software	Data was primarily analyzed using the built in Sasquatch (SQ) Software developed by Invitrogen. Histograms were created from FlowJo v11 Software of the resulting FCS files.
Cell population abundance	Cellular populations were visually inspected for GFP intensity immediately post sort using a ZOE Fluorescent Cell Imager. Purity was not calculated and relative abundance of relevant cell populations was not further determined.
Gating strategy	Cells were first gated on the area of the forward and side scatter lasers as an oval centered around the highest density of cell-like species (from around 100 - 300 AFU for both FSC and SSC) at 290 volts of the FSC and 413 volts for SSC. Cells were further sorted into single cells using FSC area and FSC height. These single cells were separated based on GFP signal into high and low GFP bins of about 50% each (dividng line at roughly 1100 AFU at 451 volts). The high GFP bin was further separated based on Dylight intensity into 5 bins which were horizontal lines of a histogram broken into roughly equal quintiles.

☐ Tick this box to confirm that a figure exemplifying the gating strategy is provided in the Supplementary Information.
