# Supplementary material for: Efficient experimental characterization of the GPCRome via deep receptor scanning
Source: Nat Commun. 2026 Aug 14;17:9796. doi: 10.1038/s41467-026-76564-7 (PMC13575135; doi:10.1038/s41467-026-76564-7)
Supplement: Supplementary file 4 [file 41467_2026_76564_MOESM7_ESM.pdf]

nature portfolio

Peer Review File

Efficient Experimental Characterization of the GPCRome via Deep Receptor Scanning

Corresponding Author: Professor Jonathan Schlebach

This file contains all reviewer reports in order by version, followed by all author rebuttals in order by version.
Editorial Note: Parts of this Peer Review File have been redacted as indicated to remove third party material where no permission to publish were obtained.

Version 0:

Reviewer comments:

Reviewer #1

(Remarks to the Author)
Summary:
This manuscript presents an ambitious deep receptor scanning platform to quantify transcript abundance, translational dynamics, and plasma membrane expression across nearly the entire human GPCRome in a pooled HEK293T system. The scale, integration of Ribo-seq with surface expression measurements, and accessible genetic library via Addgene are major strengths. However, several core design choices, most notably the universal use of N-terminal HA tags and the exclusive reliance on a single heterologous cell line, introduce important biological caveats that may confound interpretation of the retrospective ML analysis as intrinsic receptor properties. In addition, while the machine-learning analysis is informative, its conclusions remain largely descriptive without experimental validation of causality.

Major comments:

• The decision to append an N-terminal HA tag to all class A and olfactory GPCRs is a significant and potentially underappreciated perturbation. For class A GPCRs, the N terminus is not merely a passive extracellular appendage but often contains signal peptide–like or signal anchor–like information embedded within or overlapping with TM1. Inserting an HA tag upstream of the native sequence risks shifting the register or context in which TM1 is recognized. This is especially concerning in a study that explicitly interprets differences in ribosome occupancy, cotranslational dynamics, and membrane integration as intrinsic sequence constraints. Certain receptors highlighted by the field as biosynthetically fragile or unusual such as GPR37/L1, which has long been linked to signal peptide–like N-terminal behavior and ER quality control sensitivity, or chemokine receptors like CCR7 with functionally important N-terminal determinants are potential receptors for which an N-terminal tag is possibly distorting native behavior. In these cases, reduced surface expression or altered translation profiles could reflect tag-induced misrouting or folding defects rather than native limitations. Without appropriate controls, it remains difficult to disentangle genuine evolutionary or structural constraints from artifacts introduced
• A second major limitation is the exclusive reliance on HEK293T cells as the expression context. While the authors briefly acknowledge that expression "may be different in other cell lines," this caveat is not treated with the weight it deserves, given the conclusions drawn. Several of the manuscript's central observations such as ribosome pausing, disome accumulation, codon usage effects, and extreme underexpression of olfactory receptors, are highly likely to be cell-type dependent. Hundreds of olfactory receptors are evolutionarily tuned for expression in olfactory sensory neurons, not fibroblast-derived cells. GPCR folding, maturation, and ER exit are likely influenced by cell-specific chaperones, accessory proteins, lipid composition, and trafficking machinery. Olfactory receptors, in particular, are well known to require specialized cofactors (eg RTPs, REEPs) that are absent in HEK cells. Thus, the finding that olfactory receptors and certain class B/C/F receptors show translational pausing, ribosome collisions, and sequence-dependent low surface expression may say as much about HEK293T biology as about GPCR biosynthesis per se.
• The retrospective use of ML to understand feature components is a nice addition, but falls short of causal or correlative importance. The current implementation remains largely descriptive. Feature importance in a random forest or SHAP does not establish whether a given feature is causally responsible for expression differences or merely correlated with other, unmodeled properties. This is particularly relevant because many features are highly interdependent (e.g. helix hydrophobicity, packing, stability, and SASA). The authors are well positioned to go beyond retrospective inference. Even a small number of prospective validation experiments would greatly elevate the impact of this section, for example by targeted chimera experiments swapping TM6 between high- and low-expressing receptors, systematic modulation of predicted marginal TMD6 hydrophobicity within a single receptor scaffold, or testing whether ML-predicted "rescued" variants indeed

improve surface expression.

Minor comments andf questions:
• There may be an error in fig 2C - should it have been Low-UMI on the Y-axis as indicated in the text? I don't understand why this plot is anti-correlated (as mentioned in the text).
• Citing relevant literature at places with references to existing data (e.g. GnRHR known to be poorly expressed)
• in Fig. S3, The subtle decrease is not consistent (insignificant?), and does not mention the large staining intensity jump from the top two receptors to the rest. It might be better to report the AUC, and to have more receptors to be able to observe any potential trend. This small sample is insufficient to generalize across receptor classes especially given the dramatic magnitude spread in surface signal.
• Surface immunostaining assumes uniform accessibility of the HA epitope across receptors. However, N-terminal length, PTMs, and loop architecture could sterically hinder antibody binding in some receptors, independent of expression level.
• In Fig 3A/B: is the occupancy at each position an average, and should there be error representation on it? Do occupancy dips mean positions that are hard to cover for ribosomes?
• Are HEK-based transcript expression levels comparable to single cell RNA-seq data from published data such as those from the human protein atlas?
• Are all higher expressed isoforms longer than the canonical transcripts?
• Is the data in Fig. 3C corrected for transcript abundance?
• The Tet-promotor should be shown in the figure 1 diagram. Also, it was not clear to me if the GPCRs are now all under the same promotor, artificially modifying expression.
• Line 388. How are the common codons defined?
• How were the 174 'experimentally validated' GPCR splice variants chosen and why not all reported isoforms with 7 TMs
• I am wondering how consistent the "alternative splicing dramatically enhances the expression of four different receptors". No error bars means we cannot evaluate reliability here.
• It's not always clear how endogenous GPCR expression in HEK293T cells might influence ribosome profiling or proteostasis capacity, particularly for widely expressed receptors
• Yet the identification of splice variants with dramatically enhanced expression is intriguing, but the structural or biosynthetic basis for these improvements could be further explored from the ML model.
• The mentioned baseline model comparisons from the github repo are not listed in Table S1 (e.g MLP and SVM etc.)
• The definition and consistency of "high" vs "low" expression thresholds are often unclear
• While biological replicates are mentioned, the exact number of replicates contributing to certain summary plots is not always immediately clear from figure legends.
• Confidence intervals are not consistently reported alongside p-values
• Figure 4D, it is impossible to read the receptor labels
• Instead of using random splits for the 80:20% train-test splits use homology-based centroids/ sequence stratification
• Its unclear, which features specifically are AF-derived
• Does the total SASA calculation include the membrane?
• There should be a correlation matrix of the intrinsic feature correlations to guide leave-out of redundant features.
• The uses of "biosynthesis," "expression," and "plasma membrane accumulation" needs to be streamlined and differences clarified.
• The outcome on the different PME cutoffs could be helpful to report in a SF figure

(Remarks on code availability)
The readme is well written, often with more details than the methods section. THe github repo indicates sub-analyses done not mentioned in the results (supplementary).

Reviewer #2

(Remarks to the Author)
The manuscript by Tedman et al. describes the development of a platform that has the potential to support broad analyses of GPCR signalling. In this study, the authors apply the platform to systematically compare transcriptional efficiency and cell-surface expression across nearly all human GPCRs, and to examine the correlation between these properties. They further leverage machine-learning approaches to identify structural receptor features that influence cell-surface expression. Given that most work in the GPCR field relies on overexpressed receptors, the dataset presented here will be a valuable resource. In addition, the pooled plasmid library generated in this study should serve as a useful tool for the community.

I have several comments, questions, and suggestions that I believe could strengthen the manuscript; these are listed below in no particular order.

1. The authors should cross-check the signal peptides predicted by SignalP with Phobius to identify any discrepancies, particularly for receptors with notably low expression.

2. It is unclear what conclusions can be drawn from Fig. S3, as the authors employ different ligands whose agonists exhibit varying efficacies.

3. Figure 5 contains a typo in the x-axis label.

4. The authors should note that some understudied GPCRs are activated by their internal domains, such as the ECL2 region

(Discussion).

5. While the study centres on cell-surface expression, many receptors are now known to signal from intracellular compartments - especially under conditions of overexpression. Have the authors considered assessing receptor expression at different organelles?

6. The authors use native signal peptides, however, 5-HT3A and hemagglutinin are among the most commonly used non-native signal peptides in the research. It would be informative to compare native versus these two signal peptides for a subset of both low- and high-expressing receptors.

(Remarks on code availability)

Reviewer #3

(Remarks to the Author)
The authors describe a comprehensive analysis of GPCR production in HEK293 cells. After library construction, they quantitatively investigated three levels of GPCR production, transcription, translation, and surface expression, with state of the art techniques. The work gives an interesting overview of cellular GPCR production, and the experimental performance appears to be sound and reliable.

However, many results are descriptive and frequently confirm previous observations and reports. It is well known that oGPCR are problematic and have low surface expression. It is further expectable that the increasing complexity of translation and targeting/translocation is more critical for membrane protein synthesis than the initial transcription from a synthetic promoter. Folding, stability and surface expression can be more complex as they might not only depend on structural features of the GPCR. Individual GPCRs might require specific helper proteins, e.g. as it is discussed for Rtp1/oGPCRs. The current library does not consider stoichiometry of complex formation potentially necessary for efficient GPCR folding, stabilization or translocation. The results obtained from ribosome coverage could indicate problems with initiation of translation or ribosome pausing und should be better discussed. The intention of the authors was to provide a GPCR expression library to the scientific community as basis for individual research and applications. Nevertheless, in my opinion the paper would benefit from a proof of principle study to optimize the production of some identified problematic GPCRs based on the obtained results. For functional applications such as signaling or interaction studies this would be an essential prerequisite for many targets.

The discussion mostly repeats the findings and conclusions from the result section. Recombinant GPCR production has a long history, and many approaches have been published. I would thus expect a comparison of potential applications of the presented library with that of other previously published GPCR libraries. Furthermore, the results gave evidence of some frequent potential bottlenecks in GPCR production such as slow initiation of translation, ribosome pausing or inefficient translocation. The authors should discuss past or future strategies to address these restrictions with other cell-based or cell-free GPCR production pipelines.

Labels of figures 1A, 2D and E are hardly visible and should be optimized

(Remarks on code availability)

Version 1:

Reviewer comments:

Reviewer #1

(Remarks to the Author)
I thank the authors for their constructive explanation and the new CCR7 Q248I experiment. However, several of my central concerns remain only partially addressed, particularly regarding the interpretation of HA-based surface immunostaining and the extent to which the ML results can be framed as general ‘receptor-intrinsic’ determinants of GPCR expression rather than predictors of recombinant HA-tagged expression in HEK293T cells.
I appreciate the authors’ explanation that the HA tag is short and less disruptive than the larger fusion designs used in other GPCRome platforms. I also appreciate that the authors inserted the HA tag downstream of predicted signal-peptide cleavage sites for receptors with signal peptides. However, I could not find the data associated with these cleavage sites, predictions, or HA insertion constructs for the GPCRome and isoform sequences used in the study. Also, the response remains largely theoretical and does not directly test whether the N-terminal HA tag alters receptor expression, maturation, or trafficking to make broad generalizable insights into GPCR biosynthesis. This is especially important in light of the inconclusive CCR7 Q248I experiment. I therefore think this concern should be addressed either experimentally or through substantially stronger qualification of the conclusions on the ML part. A focused control panel would be sufficient and need not be GPCRome-wide. For example, the authors could compare untagged and N-terminally HA-tagged versions of selected receptors expressed from the same genomic landing pad and promoter, then measure surface expression using a validated

native extracellular antibody. Examples could include CCR7 as a receptor with important N-terminal determinants or CXCR1/CXCR2 as highlighted examples by the authors.
As it stands, the title, abstract, and discussion language still give the impression that the study reveals general sequence constraints on GPCR biosynthesis, which does not reflect the more nuanced discussion provided in the revision. I would therefore suggest that the authors consistently frame the results as identifying suggestive determinants of recombinant GPCR expression in HEK293T cells, rather than as broadly generalizable constraints on GPCR biosynthesis.
The model feature importance/ SHAP values identify features that are predictive of the measured outcome, but they do not establish that those features are causally responsible for expression differences. The new mutational experiments on CCR7 Q248I illustrate the limits of the current interpretation. The intended mutation to alter TMD6 hydrophobicity did not increase surface expression, which suggests that the importance of a feature can not as easily be extracted from the ML feature extraction, questioning the 'Reveals General Sequence Constraints on GPCR Biosynthesis' as the title claims. I would recommend substantially softening the ML conclusions throughout the manuscript interpreting the results as hypothesis-generating and suggestive rather than " our models demonstrate" or we have shown "generalizable insights into GPCR biosynthesis". Such claims would need to be supported more broadly and mechanistically for the reported top-ranked features in selected receptors. I also think the authors should include their CCR7 mutant experiment, to show the lack of direct translatability for inform construct engineering.
Its actually also unclear to me how the CCR3 glycosylation and CXCR1 ER-retention motif experiments relate to the ML analysis presented in Figure 6. The tested features (N-terminal glycosylation) are not among the SHAP-ranked predictors and hence to not support the claims made from the ML model, rather the opposite.
My remaining concern is not with the utility of the resource, which I find to be a valuable platform, but with the scope and validity of the biological claims.

(Remarks on code availability)
Already discussed during the first review.

Reviewer #2

(Remarks to the Author)
The authors have sufficiently addressed my comments to the first version of the manuscript.

(Remarks on code availability)

Reviewer #3

(Remarks to the Author)
While my comments have mostly been addressed, I remain skeptical about the value of the proposed approach. Even if suggested GPCR modifications could improve biosynthesis, they might not be compatible with functionality. The newly included application data already indicate clear limitations in the reliability of obtained predictions. Numerous related AI-based concepts are currently coming up but they unfortunately often lack support by a sufficient record of experimental data. In principle, the authors propose a prediction process but leave it up to the reader to prove its usefulness.

(Remarks on code availability)

Version 2:

Reviewer comments:

Reviewer #1

(Remarks to the Author)
I am happy with the proposed tapering of the language throughout the manuscript. In particular, I support reframing the ML findings as hypothesis-generating and suggestive determinants in HEK293T cells, rather than as evidence for broadly generalizable or causal constraints on GPCR biosynthesis.

(Remarks on code availability)

Open Access This Peer Review File is licensed under a Creative Commons Attribution 4.0 International License, which permits use, sharing, adaptation, distribution and reproduction in any medium or format, as long as you give appropriate credit to the original author(s) and the source, provide a link to the Creative Commons license, and indicate if changes were made.
In cases where reviewers are anonymous, credit should be given to 'Anonymous Referee' and the source.
The images or other third party material in this Peer Review File are included in the article's Creative Commons license, unless indicated otherwise in a credit line to the material. If material is not included in the article's Creative Commons license and your intended use is not permitted by statutory regulation or exceeds the permitted use, you will need to obtain permission directly from the copyright holder.
To view a copy of this license, visit https://creativecommons.org/licenses/by/4.0/

We are grateful for this rigorous evaluation of our findings and their interpretations concerning our development and application of an experimental GPCRome platform. Overall, we were very encouraged to see that the reviewers hold a favorable opinion of this work and believe they have done an excellent job at identifying areas that can be improved and/ or clarified for the reader. In the following, we address the reviewers' specific comments (in **black**) and outline the improvements we have made to address these concerns (in **blue**). We hope that these considerations sufficiently address any remaining questions.

REVIEWER COMMENTS

Reviewer #1 (Remarks to the Author):

Summary

This manuscript presents an ambitious deep receptor scanning platform to quantify transcript abundance, translational dynamics, and plasma membrane expression across nearly the entire human GPCRome in a pooled HEK293T system. The scale, integration of Ribo-seq with surface expression measurements, and accessible genetic library via Addgene are major strengths. However, several core design choices, most notably the universal use of N-terminal HA tags and the exclusive reliance on a single heterologous cell line, introduce important biological caveats that may confound interpretation of the retrospective ML analysis as intrinsic receptor properties. In addition, while the machine-learning analysis is informative, its conclusions remain largely descriptive without experimental validation of causality.

We thank the reviewer for this balanced perspective on our work. We appreciate the caveats raised by the reviewers, and have discussed these design choices extensively with several labs- a key reason for the extensive author list. Overall, we believe our design choices strike an optimal compromise between robustness of design and versatility. In the following, we will discuss the tradeoffs of our choices. We hope this additional information will address any lingering concerns.

Major comments

The decision to append an N-terminal HA tag to all class A and olfactory GPCRs is a significant and potentially underappreciated perturbation. For class A GPCRs, the N terminus is not merely a passive extracellular appendage but often contains signal peptide–like or signal anchor–like information embedded within or overlapping with TM1. Inserting an HA tag upstream of the native sequence risks shifting the register or context in which TM1 is recognized. This is especially concerning in a study that explicitly interprets differences in ribosome occupancy, cotranslational dynamics, and membrane integration as intrinsic sequence constraints. Certain receptors highlighted by the field as biosynthetically fragile or unusual such as GPR37/L1, which has long been linked to signal peptide–like N-terminal behavior and ER quality control sensitivity, or chemokine receptors like CCR7 with functionally important N-terminal determinants are potential receptors for which an N-terminal tag is possibly distorting native behavior. In these cases, reduced surface expression or altered translation profiles could reflect tag-induced misrouting or folding defects rather than native limitations. Without appropriate controls, it remains difficult to disentangle genuine evolutionary or structural constraints from artifacts introduced

We appreciate the reviewer's concerns, and do not take these caveats lightly. We (among others) have used this tagging strategy for other GPCRs in the past (e.g. PMID 39808594, 39078397, 32181350), but certainly agree that tagging proteins can impact their processing and expression. However, we note that this HA tag simply adds a highly polar string of 9 residues (YPYDVPDYA) to the N-terminus. Given that the SRP recognizes short strings of 8-20 hydrophobic amino acids (PMID: 10065837) within the first ~140 residues of a nascent polypeptide (PMID: 3169004), we believe this small, polar tag is generally unlikely to give rise to alternative targeting sites. Moreover, we believe the inclusion of this single tag constitutes a much smaller perturbation that is caused by the fusion domains included in the design of competing technologies. For instance, the current state-of-the-art GPCRome assay (PRESTO-Tango) employs hundreds of receptors bearing a non-native signal peptide, and N-terminal HA tag, an N-terminal FLAG, and a C-terminal V2 vasopressin receptor tail fusion domain followed by a TEV protease cleavage site and a tTA transcription factor (PMID: 25895059). Our sequence modifications are quite minimal by comparison. Moreover, inclusion of these tags opens up the doors to many other valuable experiments that require parallel immunoprecipitation.

We would humbly suggest that it is intrinsically difficult to arrive upon a one-size-fits-all tagging strategy that works equally well for 900+ different proteins. However, it is worth noting that we did include extra considerations to adjust tagging in cases where tagging would be incompatible with signal peptide processing. For instance, for

receptors that depend on signal peptides to initiate correct secretion of N-terminal domains into the ER lumen, we instead inserted the HA-tag downstream of the signal peptidase cleavage site (see Methods). We note that many such proteins exhibit considerable surface immunostaining- even in cases of certain proteins that are notoriously difficult to detect (i.e. GLP1R).

Despite these efforts, the reviewer's concerns stand. We have acknowledged this caveat explicitly in the discussion section of the revised manuscript as follows:

"*While the exogenous expression levels and cellular environment of these tagged receptors featured in these experiments is far from native and trends may be different under physiological conditions, it should be noted that these experiments could potentially be carried out in other cell lines upon introduction of single genomic attP site*."

A second major limitation is the exclusive reliance on HEK293T cells as the expression context. While the authors briefly acknowledge that expression "may be different in other cell lines," this caveat is not treated with the weight it deserves, given the conclusions drawn. Several of the manuscript's central observations such as ribosome pausing, disome accumulation, codon usage effects, and extreme underexpression of olfactory receptors, are highly likely to be cell-type dependent. Hundreds of olfactory receptors are evolutionarily tuned for expression in olfactory sensory neurons, not fibroblast-derived cells. GPCR folding, maturation, and ER exit are likely influenced by cell-specific chaperones, accessory proteins, lipid composition, and trafficking machinery. Olfactory receptors, in particular, are well known to require specialized cofactors (eg RTPs, REEPs) that are absent in HEK cells. Thus, the finding that olfactory receptors and certain class B/C/F receptors show translational pausing, ribosome collisions, and sequence-dependent low surface expression may say as much about HEK293T biology as about GPCR biosynthesis per se.

We certainly acknowledge that HEK293T cells are not universally representative of the thousands of diverse cell types that express these receptors in the human body, and apologize if our wording suggested such. However, while 293T cells may lack many cell-specific interactors they certainly contain the core machinery that mediates protein biosynthesis and quality control. For this reason, we believe the apples-to-apples comparison of receptors generated by this assay in the context of a single cell line is quite powerful for efforts to decipher common molecular features of interest. Indeed, any machine learning applications that would seek to identify these sorts of molecular signatures from analogous measurements collected under physiological conditions would potentially struggle to distinguish between differences that arise from cellular properties from those that arise from molecular variation. We suspect the use of a single cell line is key reason why our analyses identified such clear signals.

We do suspect that most of the properties identified in this analysis are potentially generalizable. And indeed, there are many molecular factors that are believed to be "generalizable" in a way that informs our understanding of GPCRs. For instance, it is widely believed that GPCRs are stabilized by sodium binding, cholesterol interactions, and palmitoylation even though these paradigms have yet to be validated across all GPCRs in an array of physiological contexts. In a way, our findings are supported by more robust, comprehensive evidence than the findings that serve as the basis of these paradigms. Regardless, we believe the beauty of this assay lies in its versatility. Should others be interested in more physiological questions, they are welcome to instead employ or compare cell lines that are more pertinent to their specific biological questions. Experimental evidence contradicting our findings in a different context could potentially lead to more interesting discoveries. For instance, if receptors with polar TMDs are highly expressible in other cell lines, it would suggest there are potentially other chaperones or cofactors that these cells rely on to overcome this common issue in GPCR biosynthesis. For these reasons, we do not see an intrinsic issue with our choice of experimental design.

The retrospective use of ML to understand feature components is a nice addition, but falls short of causal or correlative importance. The current implementation remains largely descriptive. Feature importance in a random forest or SHAP does not establish whether a given feature is causally responsible for expression differences or merely correlated with other, unmodeled properties. This is particularly relevant because many features are highly interdependent (e.g. helix hydrophobicity, packing, stability, and SASA). The authors are well positioned to go beyond retrospective inference. Even a small number of prospective validation experiments would greatly elevate the impact of this section, for example by targeted chimera experiments swapping TM6 between high- and low-expressing receptors, systematic modulation of predicted marginal TMD6 hydrophobicity within a single receptor scaffold, or testing whether ML-predicted "rescued" variants indeed improve surface expression.

We understand that our take on the ML analysis would be somewhat bold without any mechanistic investigations to point towards. However, as we noted in both the results and discussion sections, we previously investigated evolutionary modifications to the GnRHR receptor that clearly showed that small changes in the hydrophobicity of solvent-exposed TMD6 residues can have dramatic effects on surface expression. Consider the figure panels below, which are adapted from ref. 34: [redacted]

In this work, we surveyed evolutionary modifications to the mammalian GnRHR receptor, a class A GPCR, that coincided with an attenuation of its plasma membrane relative to non-mammalian versions of the receptor. Among the key differences was an uptick in the hydrophobicity of TMD6. Specifically, we identified a pair of membrane-exposed threonine residues that do not appear to form intramolecular hydrogen bonds (see panel 1). Using a biochemical membrane translocation, we confirmed that the membrane integration of TMD6 from the human GnRHR is inefficient (see panel 2, where G2 > G1). Moreover, reverting these positions to the hydrophobic side chains that are common in non-mammalian GnRHRs enhances the membrane integration (see panel 2, G1 > G2) and, in the case of T227I, also restores the plasma membrane expression of the receptor. In conjunction with previous biological evidence, our previous findings suggested that evolutionary modifications to the hydrophobicity had compromised the efficiency of receptor biosynthesis in order to downregulate the GnRH signaling pathway (see Ref. 37 in the revised manuscript).

Following these investigations, it was unclear whether our observations only pertained to GnRHR or if they were instead suggestive of a more general biosynthetic bottleneck in GPCR biosynthesis. Needless to say, we were pleasantly surprised to find that the hydrophobicity of TMD6 is the strongest marker for receptor expression levels. We note that this conclusion is obvious even without the results of the random forest models or SHAP analysis (see Fig. 6D). For these reasons, we feel quite confident that the importance of certain features is likely general even though the reviewers point related to correlations between features is certainly valid. In essence, we already completed the proof of principle investigations requested by the reviewer years ago.

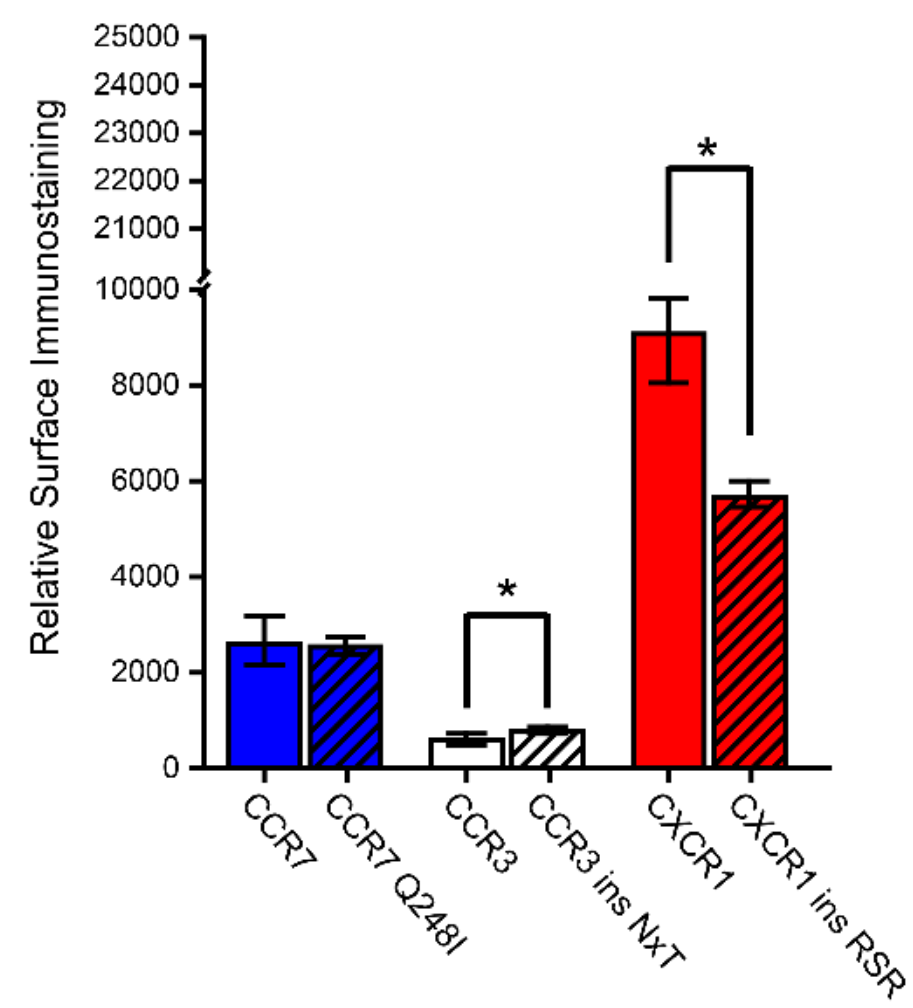

In light of the reviewer's comments, we carried out additional experiments to assess whether our discoveries are sufficient to rationally improve GPCR expression based on differences in the sequences of related receptors with divergent expression. To this end, we analyzed differences between the features of various chemokine receptor family to identify modifications that may help increase or decrease the expression of certain receptors. Despite considerable sequence identity, we identified sizable differences between the plasma membrane expression of CCR3 (low), CCR7 (intermediate), and CXCR1 (high). We note that some of the key features identified in our machine learning analysis such as SASA are impossible to modify without perturbing other relevant features. We therefore focused on smaller differences between these receptors that could be modified with a small number of mutations (i.e. TM6 hydrophobicity) as well as certain regulatory motifs that are obviously distinct. We first attempted to enhance the expression of CCR7, which features a marginally hydrophobic TMD6, by reverting one of its polar residues to a hydrophobic side chains (Q248I). Unlike GnRHR (see above), in this case this substitution did not result in a measurable increase in surface expression (see graph on the right). In this case, it may be that changes in the hydrophobicity of this domain are insufficient to overcome the destabilizing impacts of other features. For instance, CCCR7 also contains an extremely long ECL3 (30 residues, 97th percentile). It is possible that, even with improved TMD6 hydrophobicity,

the excessive length of this loop prevents rescue of expression. It is fundamentally difficult to know which of these features is limiting in the absence of other contextual information.

Through our inspection of these sequences we also noticed inconsistencies associated with various sequence motifs. For instance, the poorly-expressed CCR3 receptor lacks a consensus N-terminal glycosylation site whereas the highly-expressed CXCR1 protein features a sub-optimal version of an C-terminal RxR motif, which has been found to promote the ER retention of other proteins (PMID: 15805225). We therefore generated mutant versions of each receptors containing these motifs then measured their relative surface immunostaining by flow cytometry. Our results reveal that insertion of a consensus glycosylation site into the N-terminal loop of CCR3 (an insertion of a asparagine at residue 2) results in a small yet statistically significant increase in surface expression (see above). In contrast, incorporation of the RSR ER retention motif into CXCR1 (H334S) significantly reduced its plasma membrane expression (see above). Together, these results suggest comparative analyses of GPCR features and expression profiles could potentially be used to guide protein engineering efforts, though this would likely require the development of a more specialized prediction platform. We note that such applications are beyond the scope of these investigations.

Minor comments and questions:

There may be an error in fig 2C - should it have been Low-UMI on the Y-axis as indicated in the text? I don't understand why this plot is anti-correlated (as mentioned in the text).

We thank the reviewer for their careful evaluation of our figures. The original figures were correct- Figure 2C showed that the enrichment of certain transcripts in the high-GFP subpopulation is correlated with the relative abundance of the transcript overall (i.e. total RNA-Seq reads). Nevertheless, we now see that we were inconsistent in the way that we cast the axes in Figures 2B and 2C. We have now cast all figures in the manuscript in terms of the enrichment of receptors within the high-GFP population for the sake of consistency (see updated panels 2A, 5Am and Doc. S1). The figures now clearly show that class A, B, C, and F receptors all tend to be enriched within the high-GFP population, which suggests their transcripts exhibit higher stability than those encoding olfactory and taste receptors in the context of HEK293T cells. We apologize for any confusion this may have caused.

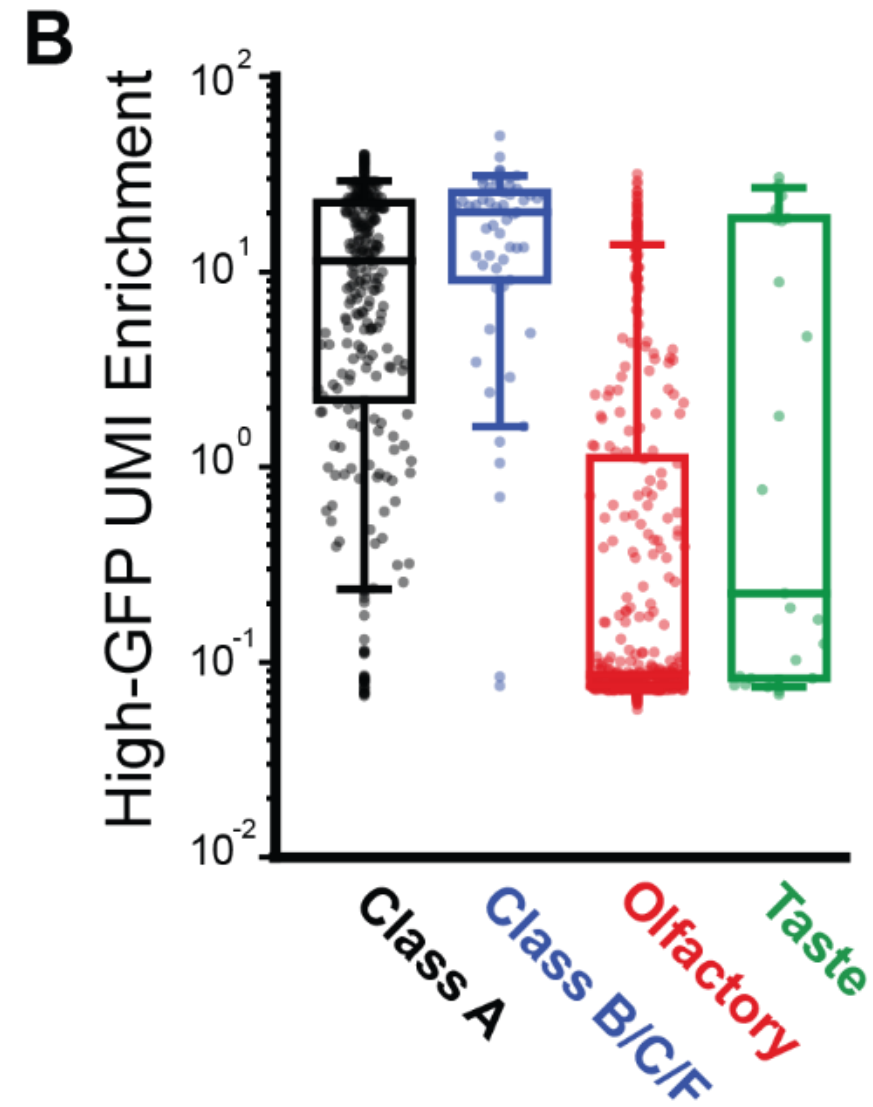

Citing relevant literature at places with references to existing data (e.g. GnRHR known to be poorly expressed)

We apologize for this oversight, and have added a new reference to support this claim (see reference 27 in the revised manuscript).

• in Fig. S3, The subtle decrease is not consistent (insignificant?), and does not mention the large staining intensity jump from the top two receptors to the rest. It might be better to report the AUC, and to have more receptors to be able to observe any potential trend. This small sample is insufficient to generalize across receptor classes especially given the dramatic magnitude spread in surface signal.

We agree that this small sample of receptors is insufficient to generalize. But absent a scalable high throughput activity assay, there is currently no way to make head-to-head comparisons across the entire collection of receptors. We are currently working to develop such approaches, though we believe this constitutes a technical challenge that lies beyond the scope of this investigation. Moreover, even if we had such an assay, we would not have access to the hundreds of distinct agonists that would be needed to activate all the diverse receptors within the pool. Regardless, we note that the pilot experiments in Fig. S3 were never intended to establish that all the expressed receptors were active. Rather, these experiments were carried out to discern whether there was reason to believe that poorly expressed receptors were unlikely to signal. The results clearly show low GPCR abundance does not preclude signaling. We also note that we did not design these experiments with the intent to compare the magnitude of the downstream calcium flux. Indeed, the fluorescent signal generated by the FURA Red assay is a relative measurement, and a comparison of absolute intensities (or the areas under the curves) is likely to be qualitative at best. We have adjusted the wording of the section to better reflect the nature of this comparison as follows:

“*While there may be subtle differences in the magnitude of the signaling responses generated by low-abundance receptors, we observe activation signals that are well above baseline in all cases (Fig. S3)*.”

Surface immunostaining assumes uniform accessibility of the HA epitope across receptors. However, N-terminal length, PTMs, and loop architecture could sterically hinder antibody binding in some receptors, independent of expression level.

We accept and acknowledge this caveat. We have noted this limitation with the following text in the revised discussion:

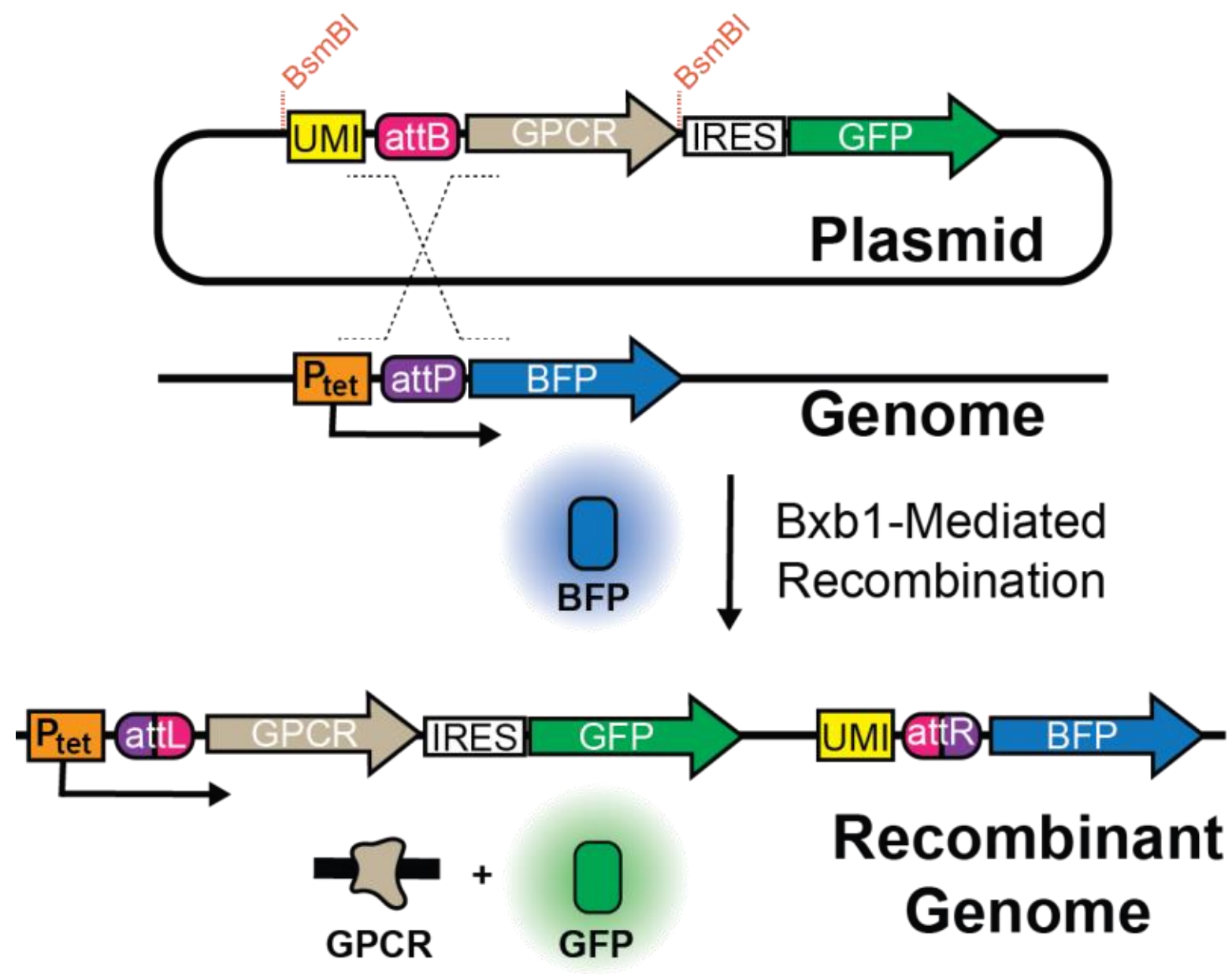

“*For instance, proteomic mass spectrometry could be used to track the differential chemical modification via co-immunoprecipitation of hundreds of receptors in parallel, though it is important to note that the N-terminal HA tag may not be uniformly accessible across the receptor pool*.”

In Fig 3A/B: is the occupancy at each position an average, and should there be error representation on it? Do occupancy dips mean positions that are hard to cover for ribosomes?

The occupancy plotted in Figure 3 do not reflect any sort of averaging across transcripts. Rather, they effectively depict the number of protected reads at each position per million nucleotides collected in the deep sequencing data. This is a fairly standard transformation of Ribo-Seq data, and because it is simply a scaled read count, we do not feel it is appropriate to include any sort of uncertainty metric. Dips in the occupancy simply reflect positions we see fewer reads, which could arise for a number of reasons. We believe we were careful not to over-interpret the data given their semi-empirical nature.

Are HEK-based transcript expression levels comparable to single cell RNA-seq data from published data such as those from the human protein atlas?

Given that the receptors in our pool are uniformly transcribed via an exogenous Tet-On promoter, we have refrained from any systematic comparisons to native transcript levels. In the event of an apparent correlation, it would not be clear what to conclude given the heterogeneous nature of single-cell RNA-seq data from different cell lines. Moreover, apparent variations in single cell/ endogenous expression likely reflect a composite measure of both differences in transcriptional output and transcript degradation in the cell. In contrast, given the minimal variation in the transcriptional output from our genomic promoter system, we can interpret our observations in terms of transcript stability alone (see below for additional details).

Are all higher expressed isoforms longer than the canonical transcripts?

No- some of the higher expressed isoforms are actually shorter than the canonical receptors. This is, in fact, the case for the two isoforms with the largest increases in expression (isoforms of ADGRE3 and GPR132). Overall, there is no apparent correlation between isoform size and expression changes.

Is the data in Fig. 3C corrected for transcript abundance?

We apologize if this was unclear, but the transcript abundance is actually on the x-coordinate (RNA Seq Reads). We simply plotted the reads for each receptor transcript in terms of their Z-score in order to depict statistical variations in transcript abundance.

The Tet-promotor should be shown in the figure 1 diagram. Also, it was not clear to me if the GPCRs are now all under the same promotor, artificially modifying expression.

We apologize for the confusion, which potentially stems from the condensed size of Figure 1A in the manuscript draft. The transcription of each GPCRs in our assay is driven by the same genomic Tet-On promoter. The uniform genomic context and induction treatment normalizes the transcriptional output for each GPCR under the

experimental conditions. This consistency provides a key rationale for our interpretation of transcript and protein levels without any additional layers of complication related to endogenous regulation of transcription. We have made Figure 1A larger for readability purposes and highlighted the key characteristics of our design (see the revised graphic on the right).

Line 388. How are the common codons defined?

We apologize if this was unclear, but given the length of the manuscript, we thought it would be best to include this detail in the *Materials and Methods* section. In short, codons were designated as "common" or "rare" based on the %MinMax algorithm in a manner that reflects their relative usage in the context of the human genome. The manuscript contains the following technical description of these definitions:

"*To analyze each coding sequence, the fraction of the sequence using rare or common codons was calculated as the fraction of sequence with %MinMax <0 or %MinMax >0, respectively. Codon usage data for H. sapiens was sourced from the HIVE-CUT database.*[43,44]"

How were the 174 'experimentally validated' GPCR splice variants chosen and why not all reported isoforms with 7 TMs

These alternative transcripts were previously validated by cross referencing RNA-Seq data from the Genotype–Tissue Expression (GTEx) Consortium with sequence/ structural information from GPCRdb (see Ref. 21 in the revised manuscript). We chose these due to the experimental evidence suggesting their transcripts accumulate in certain tissues. Expanding the library to incorporating a wider array of putative splice variants would have been prohibitively expensive. We now see that we should have cited this reference earlier in the manuscript when we introduced these isoforms. We have rearranged the references and have added this citation where we first discuss these isoforms (see line 110).

I am wondering how consistent the "alternative splicing dramatically enhances the expression of four different receptors". No error bars means we cannot evaluate reliability here.

We apologize for this oversight and have included the error bars depicting the standard deviation across three biological replicates for both panels in Figure 5 (shown on the right). All four of these receptors exhibit statistically significant increases in expression relative to their corresponding canonical receptors (students t-test, $p < 0.005$).

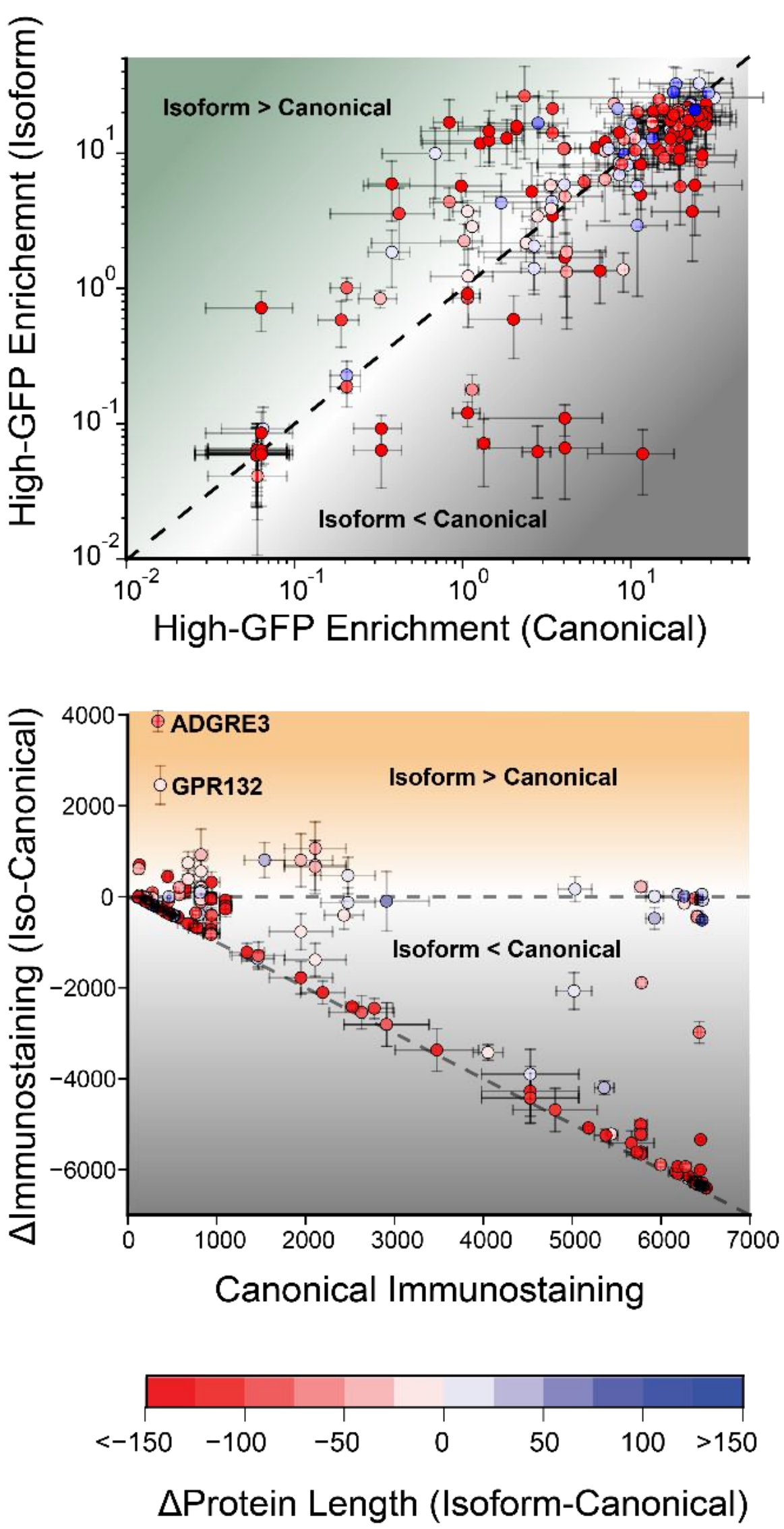

It's not always clear how endogenous GPCR expression in HEK293T cells might influence ribosome profiling or proteostasis capacity, particularly for widely expressed receptors

We do not believe that endogenously expressed transcripts should impact our RiboSeq analyses because both the endogenous and exogenous transcripts should share the same sequence and therefore exhibit similar translational activity. This is a key reason why we opted to preserve the native codon usage rather than employ codon-optimized transcripts. Regardless, we note that we primarily report a meta-analysis of whole classes of GPCRs and have de-emphasized the analysis of individual transcripts. This analysis dilutes ribosome-protected fragments from endogenous transcripts into the wider set of ribosome-protected fragments from the library. We see no need to differentiate based on the transcriptional source of the transcript.

Yet the identification of splice variants with dramatically enhanced expression is intriguing, but the structural or biosynthetic basis for these improvements could be further explored from the ML model.

We agree that the molecular basis of these observations is intriguing. Based on the location of the modifications caused by these splicing events, we suspect they impact the glycosylation of immature receptors and the manner in which they are sorted within the ER. I thorough investigation of the changes in the modification and sorting of these isoforms would be interesting, but would significantly expand the scope of these investigations in a manner that may detract from the key focus of this work. Indeed, we believe we have disclosed many observations that merit additional investigations, but humbly suggest that following up on each of these discoveries lies beyond the scope of this specific investigation.

The mentioned baseline model comparisons from the github repo are not listed in Table S1 (e.g MLP and SVM etc.)]

We thank the reviewer for pointing out this accidental omission. We have now added this information to the revised version Table S1.

The definition and consistency of “high” vs “low” expression thresholds are often unclear

We appreciate the feedback and acknowledge the categorizations of “high” and “low” for expression is inherently somewhat arbitrary. The difficulty lies in the fact that the fluorescence patterns are inherently relativistic and there are no relevant metrics (ie. a wild type) by which we can gauge a “high” and “low” expression level outside of the GPCR population itself. Since both of our distributions are bimodal, we grouped the receptors based on which distribution they primarily fell into by representing this cutoff at a value near the local minimum (about 100 RFU for GFP and 1000 RFU for the immunostaining intensity. The immunostaining division was further correlated to the expression of two outside controls which were previously known to express very well (rhodopsin) and very poorly (GNRHR). Furthermore, the “high” and “low” thresholds can be dependent on the gating strategy. Our gating strategy is depicted on the GFP histogram, while we provide a depiction of our gating strategy for the surface immunostaining intensity in the supplement. (Austin)

While biological replicates are mentioned, the exact number of replicates contributing to certain summary plots is not always immediately clear from figure legends.

We apologize for failing to state this more clearly. All receptor scanning measurements were carried out with three biological replicates. To clarify this point, we have added this information to the figure legends, where appropriate.

Confidence intervals are not consistently reported alongside p-values

In the revised draft, we have attempted to provide more robust confidence estimates, where appropriate. For our Spearman’s rank correlations, we have included confidence intervals for the ρ value. Upon further consideration, we opted to report a false discovery rate based on a Quasi-Likelihood F-test, as this is a commonly employed metric in the evaluation of RNA-seq data and various other comparable experiments. Please note that this confidence metric is different for each receptor (see Doc. S1).

Figure 4D, it is impossible to read the receptor labels

We thank the reviewer for this feedback. Indeed, we struggled with various approaches to depict the phylogenetic variance in expression. Unfortunately, we believe that this wheel remains the best option. Nevertheless, we do believe this is still useful for those interested in specific classes of receptors, as they can zoom in on digital versions of the manuscript. We will pay close attention to this aspect of the manuscripts during review of the galley proofs, should this article be accepted. Perhaps we can provide a higher resolution version of this figure for the final article.

Instead of using random splits for the 80:20% train-test splits use homology-based centroids/sequence stratification

We thank the reviewer for this suggestion, but note that there is little reason to believe that our models are identifying phylogenetic trends given the nature of the training data (see Fig. 4D). Nevertheless, to explore how this approach may impact model performance, we trained new random forest models using homology-aware train/ test splits with MMseqs2 sequence clustering. GPCR protein sequences were clustered at two different sequence identity thresholds (50% and 90%) and all members of each cluster were assigned exclusively to either the training or testing set using scikit-learn’s GroupShuffleSplit (50 iterations, 80:20 train/test ratio). This ensures that no closely related receptor pair spans both sets, preventing data leakage through sequence homology. As we expected, model performance decreases modestly under homology-aware evaluation relative to standard

random splitting. For the random forest model trained on all GPCRs with the full feature set, AUROC decreases from 0.818 ± 0.025 (random split) to 0.784 ± 0.065 (homology-aware, 50% identity clustering, see ROC plot to the right). For the model trained on high-GFP (i.e. stable transcript) GPCRs using structural and topological features, AUROC decreases from 0.752 ± 0.048 to 0.684 ± 0.067. Performance is robust across clustering thresholds: at 60% identity (473 clusters) the all-features model achieves AUROC = 0.785 ± 0.039, while even at the most stringent threshold tested (40% identity, 231 clusters, largest cluster = 261 receptors), the model retains an AUROC of 0.686 ± 0.147, well above random chance.

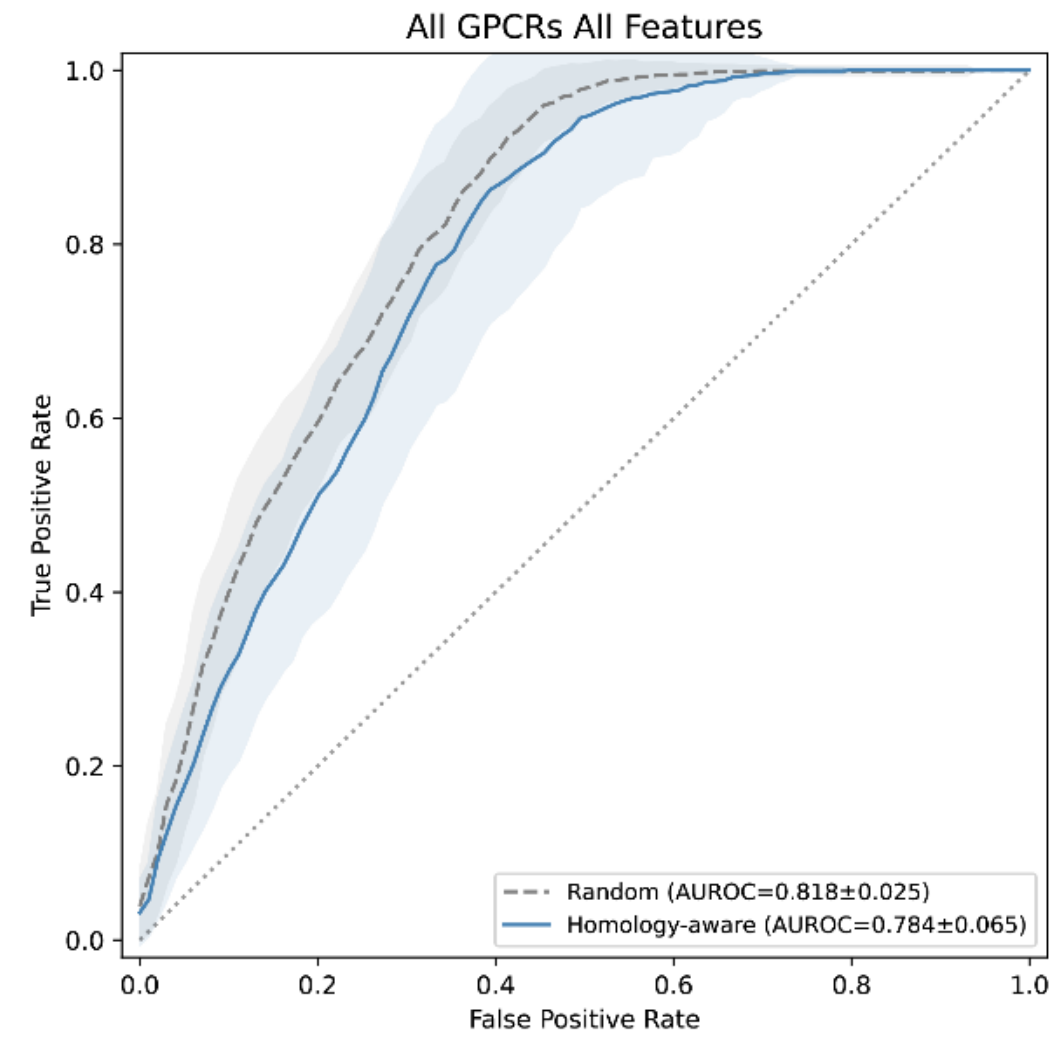

Importantly, the relative ranking of model configurations is preserved under homology-aware evaluation. Models incorporating both structural and topological features continue to outperform those using either feature set alone, and the top-performing features identified by SHAP analysis — including the predicted free energy of membrane insertion for TMD6 ($\Delta G_{app,pred}$) and total SASA — remain the most predictive across all clustering thresholds (see SHAP plot to the right). These results support the conclusion that these biophysical constraints on GPCR expression are generalizable across diverse receptor subfamilies and are not artifacts of phylogenetic similarity between training and testing receptors.

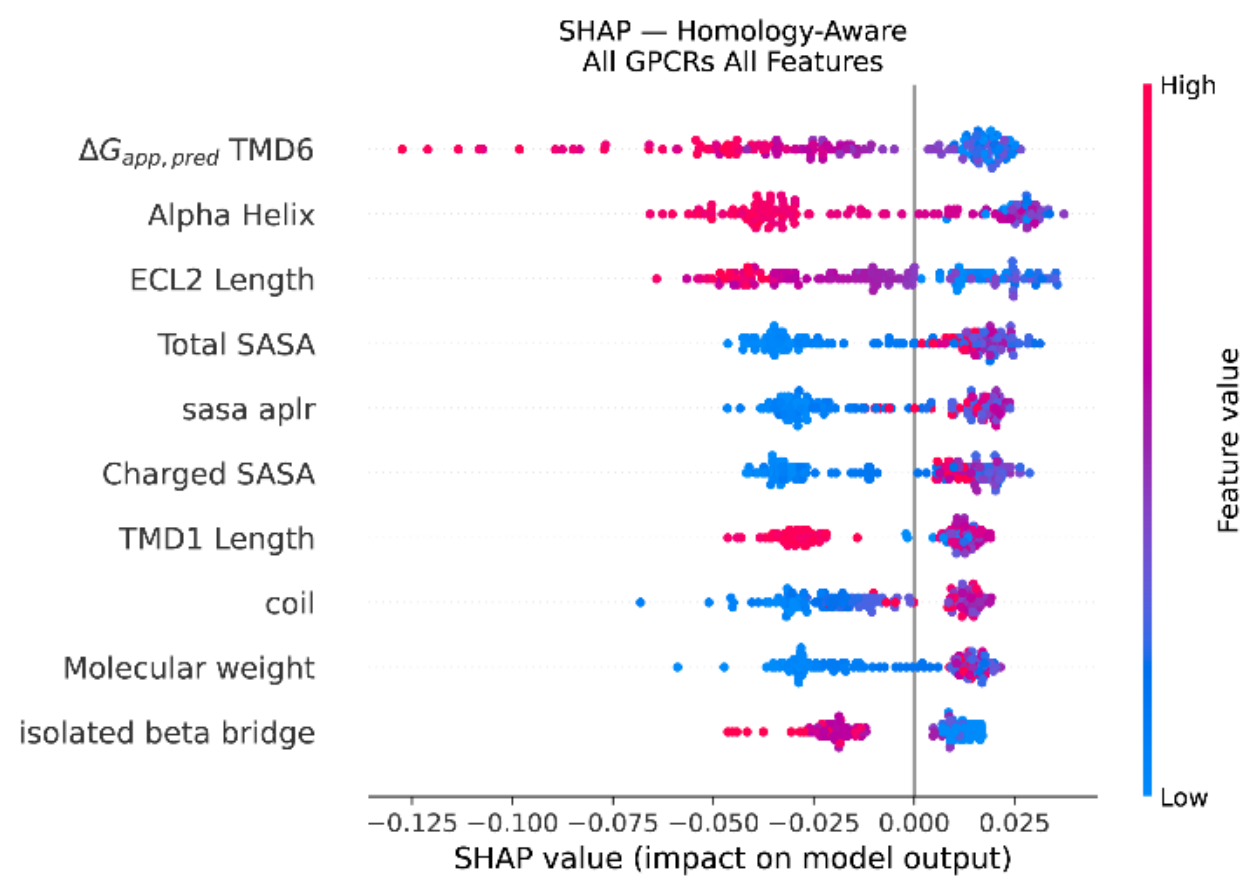

After evaluating both approaches to splitting and training the test sets, we believe the homology-aware approach to clustering requires arbitrary selection of a threshold for identity clustering. Given that the differences in performances are immaterial, we opted to retain our original random 80: 20 approach to model training for the sake of simplicity. Nevertheless, in our efforts to rigorously compare these models, we found conclusions drawn from an ensemble of classifier models to be more robust. For this reason, we opted to replace the metrics and plots derived from a single model with those instead derived from a set of 50 models that were iteratively trained using distinct test: train sets. We also corrected a small error in the calculation of our length feature that had a small impact on receptors containing signal peptides. The revised version of Figure 6 therefore contains both refined UMAP and ROC plots. Table S1 now reports averaged metrics across 50 models. We note that the SHAPLEY analysis for these aggregate models are slightly different than the originals. While the hydrophobicity of TMD6 is still the top-performing feature, total SASA is not ranked as highly. For this reason, we have adjusted Figure 6 and the corresponding text to instead feature differences in α-helical content.

Its unclear, which features specifically are AF-derived

We have added this information to the revised version of Table S3.

Does the total SASA calculation include the membrane?

No, the SASA calculations reflect the total surface area of the protein that is exposed to both the membrane and to water. We considered trying to divide the SASA calculations according to which parts of the protein are exposed to the membrane, but ultimately decided this would involve considerable uncertainty, as the manner in which these proteins are embedded in the membrane is unclear.

There should be a correlation matrix of the intrinsic feature correlations to guide leave-out of redundant features.

We thank the reviewer for this suggestion and have added the requested correlation matrix to Figure S5 in the revised version of the supplement.

The uses of “biosynthesis,” “expression,” and “plasma membrane accumulation” needs to be streamlined and differences clarified.

We thank the reviewer for this suggestion and have taken made sure to consistently use expression in place of accumulation. We have also replaced "biosynthesis" with "cotranslational assembly" at points in the manuscript to be more specific. The remaining usages of "biosynthesis" refer to collective processes that include transcription and translation.

The outcome on the different PME cutoffs could be helpful to report in a SF figure

We thank the reviewer for this suggestion and have added a new supplementary figure showing the relationship between the PME cutoff and the AUROC in Fig. S6 in the revised version of the manuscript.

Reviewer #1 (Remarks on code availability):

The readme is well written, often with more details than the methods section. The github repo indicates sub-analyses done not mentioned in the results (supplementary).

We thank the reviewer for their careful evaluation of our GitHub repository, which we took great care in curating. We believe it is important to be transparent for those that may have other questions regarding potential ways to analyze these data. Nevertheless, we opted not to add more discussion related to the finer points of machine learning so as not to distract from the key points related to the anatomy of the GPCRome. For this reason, we have chosen to leave the additional materials on GitHub without modifying the manuscript further.

Reviewer #2 (Remarks to the Author):

The manuscript by Tedman et al. describes the development of a platform that has the potential to support broad analyses of GPCR signaling. In this study, the authors apply the platform to systematically compare transcriptional efficiency and cell-surface expression across nearly all human GPCRs, and to examine the correlation between these properties. They further leverage machine-learning approaches to identify structural receptor features that influence cell-surface expression. Given that most work in the GPCR field relies on overexpressed receptors, the dataset presented here will be a valuable resource. In addition, the pooled plasmid library generated in this study should serve as a useful tool for the community.

We thank the reviewer for their careful reading of the manuscript. We share the reviewer's optimism regarding the broad utility of this library and workflow.

I have several comments, questions, and suggestions that I believe could strengthen the manuscript; these are listed below in no particular order.

1. The authors should cross-check the signal peptides predicted by SignalP with Phobius to identify any discrepancies, particularly for receptors with notably low expression.

This is a reasonable request, though we note that SignalP 6.0 is a more modern, specialized language model that can predict all five types of signal peptides with higher accuracy than Phobius (PMID: 34980915). The ability to discern between *bona fide* signal peptides and N-terminal transmembrane domains is also of key importance for our designs, which is a key issue with Phobius. Moreover, Phobius does not predict the specific position of the signal peptidase cleavage site, which is an important piece of information to ensure any inserted epitope tags are not proteolytically cleaved. The difference in the predictive outputs is best illustrated by through head-to-head comparisons of their predictions. To evaluate differences in these predictions more broadly, we used Phobius to generate signal peptide predictions for each receptor in our collection then compared its output to those of SignalP 6.0.

Overall, the two methods exhibit 96.3% agreement (738/766) with respect to the identity of transcripts that contain signal peptides. This includes 63 receptors predicted to contain signal peptides by both tools and 675 predicted to lack them. Nevertheless, there are 28 transcripts with divergent predictions, including five receptors where only SignalP predicts a signal peptide (RXFP1, RXFP2, ADGRG2, FZD4, and GRM8). Notably, two of these receptors (FZD4 and GRM8) exhibit high surface expression (PME = 5831 and 6468, respectively), indicating the HA placement did not compromise their biosynthesis. The low expression of RXFP1 and RXFP2 is more likely attributable to the well-documented structural complexity of their large leucine-rich repeat ectodomains that can present considerable folding challenges (PMID: 37081311, 17293890, and 26441827).

There were also 23 receptors that were predicted to contain a signal peptide by Phobius but not SignalP 6.0. The majority of these cases (14/23) involve olfactory receptors, which is consistent with the well-documented tendency of hidden Markov model-based topology predictors to misclassify the first transmembrane domain of

N_{out} oriented membrane proteins (PMID: 15111065 and 17483518). Among the non-olfactory receptors in this set, we note that CRHR2, which is a class B GPCR that could potentially harbor a signal peptide, was classified by SignalP as lacking one. This is consistent with the fact that the CRHR2α isoform contains a unique pseudo signal peptide that is not cleaved from the mature receptor (PDIB: 9717834 and 16766521). We note that this receptor achieves moderate expression (PME = 2385) despite receiving an N-terminal HA tag. Overall, there is no statistically significant association between the surface immunostaining of receptors for which the two predictors disagree (Mann-Whitney $p = 0.51$; median PME = 826 for discrepant receptors vs. 876 for concordant receptors). This result is robust when restricting the analysis to the subset of 385 receptors with stable transcripts that are not enriched within the low-GFP subpopulation (Mann-Whitney $p = 0.52$), as well as to the 381 receptors with unstable transcripts ($p = 0.41$), confirming that transcript-level variation does not confound this comparison. We also found no significant association between prediction discrepancy and transcript abundance (RNA-Seq RPKM, $p = 0.59$), GFP enrichment ($p = 0.70$), GC content ($p = 0.35$), or protein length ($p = 0.10$). Taken together, these analyses confirm that any inaccuracies in signal peptide predictions are unlikely to represent a key factor influencing variations in receptor expression.

2. It is unclear what conclusions can be drawn from Fig. S3, as the authors employ different ligands whose agonists exhibit varying efficacies.

As noted above, we agree that these results are not comprehensive in the same way our receptor scanning measurements are. The key rationale for these experiments concerned the nuanced differences in the design of this library relative to PRESTO-Tango, which is the current standard for high-throughput GPCR profiling. Specifically, PRESTO-Tango employs non-native signal peptides and codon optimized transcripts to achieve high expression of each receptor in order to ensure detectable signaling outputs. By comparison, we opted to preserve as many native features of the transcripts and receptors as possible, which facilitated many of the discoveries related to transcript stability and translational output detailed herein. Based on this aspect of our design, we were concerned that that the low expression of some receptors may preclude their functional characterization using this library. We therefore carried out these limited studies to assess whether receptors with undetectable surface expression could still potentially generate s measurable signaling response. While the limited number of measurements in Figure S3 prevents us from generalizing our findings, these data at least provide anecdotal evidence that low surface expression does not preclude functional characterization. We were careful not to overstate our interpretation of these findings. We hope this clarifies this matter for the reviewers.

3. Figure 5 contains a typo in the x-axis label.

We thank the reviewer for their careful evaluation and for noticing this error. We have fixed this typographical error in the revised manuscript.

4. The authors should note that some understudied GPCRs are activated by their internal domains, such as the ECL2 region (Discussion).

We thank the reviewer for this suggestion. We have modified the discussion to include this important information:

“Interestingly, our machine learning models suggest the three most important topological features are the lengths of the N-terminus and extracellular loop 2 (ECL2), as well as the hydrophobicity of TMD6, all of which are dynamically rewired during GPCR activation. Indeed, while both the N-terminus and ECL2 mediate agonist binding, while in some niche cases ECL2 acts as an internal agonist mediating receptor signaling.[36]”

5. While the study centres on cell-surface expression, many receptors are now known to signal from intracellular compartments - especially under conditions of overexpression. Have the authors considered assessing receptor expression at different organelles?

This is an excellent point and would be highly interesting to explore. However, quantifying sub-cellular localization typically requires intracellular immunostaining and colocalization measurements. Unfortunately, this requires the cells to be permeabilized and fixed. These sorts of steps are fundamentally incompatible with the Sort-Seq methodologies used in receptor scanning, as the cells must be re-grown after sorting. Tracking intracellular signaling events will require the development of specialized assays that enable signaling events from different organelles generate distinct fluorescent signals. Though this is clearly beyond the scope of this work, we will continue to think about how we could work towards this goal.

6. The authors use native signal peptides, however, 5-HT3A and hemagglutinin are among the most commonly used non-native signal peptides in the research. It would be informative to compare native versus these two signal peptides for a subset of both low- and high-expressing receptors.

It is unclear how we could universally append distinct signal peptides given the heterogeneous nature of the signaling sequences across the GPCR family. For instance, TMD1 serves the signal peptide for class A receptors. Any non-native signal peptides appended to these receptors would have to adopt an N_{in} orientation to avoid inverting the topology of these receptors. In contrast receptors bearing large extracellular domains (i.e. class B, C, and F) have their own native N_{in} signal peptides. Any additional signal peptides appended to these receptors would therefore need to adopt the opposite N_{out} topology. While we designed the library so that it could be moved from one vector to another, extensive modifications to the signal peptides cannot be achieved in parallel and would require the preparation of hundreds of plasmids. Moreover, focused efforts to determine the effects of non-native signal peptides on select receptors with low expression would carry the same caveat the reviewer raised about our targeted functional analyses in Figure S3. For these reasons, we opted not to carry out additional investigations into the effects of non-native signal peptides on receptor expression.

Reviewer #3 (Remarks to the Author):

The authors describe a comprehensive analysis of GPCR production in HEK293 cells. After library construction, they quantitatively investigated three levels of GPCR production, transcription, translation, and surface expression, with state of the art techniques. The work gives an interesting overview of cellular GPCR production, and the experimental performance appears to be sound and reliable.

We thank the reviewer for this balanced overview of our work.

However, many results are descriptive and frequently confirm previous observations and reports. It is well known that oGPCR are problematic and have low surface expression.

We acknowledge that many of our general observations confirm that a wide swath of the GPCR family conform to certain widely held beliefs, most of which are based on anecdotal observations of a limited number of well-characterized receptors. However, there are clearly exceptions to every one of these general trends. We simply chose not to dwell on the outliers for the sake of clarity. Our data provide a highly useful resource to identify the exceptions to these "rules" which will provide new opportunities to explore new aspects of GPCR biology. For instance, as the reviewer notes, *most* olfactory receptors exhibit undetectable surface expression. However, for those interested in the structural characterization of olfactory receptors, our analysis identifies over 80 olfactory receptors that achieve moderate expression levels (immunostaining intensity >1,000). As a specific example, we note that the immunostaining of the olfactory receptor with the highest expression (OR10K2) is 20-fold higher than the lowest expressing olfactory receptor (OR2J2). Based on these considerations, we believe the utility of these data go far beyond simply serving as evidence supporting the conclusions we draw herein.

It is further expectable that the increasing complexity of translation and targeting/translocation is more critical for membrane protein synthesis than the initial transcription from a synthetic promoter. Folding, stability and surface expression can be more complex as they might not only depend on structural features of the GPCR.

Yes, we agree in general terms, but again note that our data reveal plenty of exceptions that can be leveraged for new discoveries. For instance, while it is widely believed that "translational efficiency" is important for protein expression, we identify a variety of receptors that appear to have high translational efficiency, but low expression (and vice versa). Our systematic characterization of the whole protein family will provide clear sets of receptors that can be used to design comparative studies.

Individual GPCRs might require specific helper proteins, e.g. as it is discussed for Rtp1/oGPCRs. The current library does not consider stoichiometry of complex formation potentially necessary for efficient GPCR folding, stabilization or translocation.

We agree, but note that this platform can easily be employed to survey the specificity of any "helper proteins" of interest. For reference, our library is compatible with a recently described cell line that would allow us to recombinantly express the GPCRome in combination with any additional protein of interest (see doi: 10.1021/acssynbio.3c00355). Similarly, our receptor scanning approach can be combined with CRISPR to survey how individual receptors depend on endogenous proteins of interest (see doi: 10.7554/eLife.107180.3). Thus, the versatility of this experimental platform will empower other systems-wide approaches to explore these aspects of GPCR biology.

The results obtained from ribosome coverage could indicate problems with initiation of translation or ribosome pausing und should be better discussed.

We thank the reviewer for raising this point. To highlight this possibility, we have added the following text to the revised discussion section:

"*These features also alter where ribosomes accumulate within transcripts (Fig. 3 A-B), which could influence the cotranslational assembly of nascent receptors. Ribo-Seq data suggest ribosomes tend to accumulate near the 5' of these transcripts, which is suggestive of sub-optimal translation initiation. This observation potentially suggests native 5' UTRs may help tune physiological translation efficiencies*."

The intention of the authors was to provide a GPCR expression library to the scientific community as basis for individual research and applications.

We note that this is one of many intentions. We believe this resource will ultimately enable a wide variety of biochemistry and cellular biology applications that extend far beyond what we can currently imagine.

Nevertheless, in my opinion the paper would benefit from a proof of principle study to optimize the production of some identified problematic GPCRs based on the obtained results. For functional applications such as signaling or interaction studies this would be an essential prerequisite for many targets.

We thank the reviewer for this input. As part of this revision, we attempted to apply our findings to optimize the expression of a few receptors, with mixed results (see response to Reviewer 1). One of the challenges associated with our findings is that the random forest models identify combinations of features that are associated with high expression. Thus, in some cases, the improvement of one feature may not translate to higher protein levels if there is also a secondary factor that also limits biosynthesis, folding, and/ or trafficking. For instance, we previously showed small changes in the hydrophobicity of TMD6 is sufficient to quadruple the expression of GnRHR (see Ref. 34). But this same modification may not generate the same outcome in the context of a receptor that also has a short C-terminal loop. Nevertheless, we note that the development of an algorithm to improve GPCR expression was not the focus of this investigation. Given the broad scope of this manuscript, we feel this is best left for subsequent investigations.

The discussion mostly repeats the findings and conclusions from the result section. Recombinant GPCR production has a long history, and many approaches have been published. I would thus expect a comparison of potential applications of the presented library with that of other previously published GPCR libraries. Furthermore, the results gave evidence of some frequent potential bottlenecks in GPCR production such as slow initiation of translation, ribosome pausing or inefficient translocation. The authors should discuss past or future strategies to address these restrictions with other cell-based or cell-free GPCR production pipelines.

We certainly appreciate the reviewer's point, but note that we have intentionally avoided over-emphasizing applications related to the optimization of GPCR expression. Though expression is certainly important, it is not clear to us whether expression levels in HEK293T cells is particularly relevant to expression levels in prevailing expression systems (i.e. insect cell culture). Furthermore, we believe much of this platform's utility lies in its potential to facilitate scalable signaling measurements across the GPCRome. For this reason, we chose to emphasize comparisons to other high-throughput GPCR pharmacology assays such as PRESTO-Tango, which constitute the most obvious point of comparison (see the second paragraph of the discussion).

Labels of figures 1A, 2D and E are hardly visible and should be optimized

We thank the reviewer for the feedback. We have adjusted the labels for 1A, 2D and 2E for improved readability.

We are grateful for the additional evaluation of our findings and their interpretations. We appreciate the reviewers' concerns related to the scope of our claims and have made several amendments to the text in order to ensure we have not overstated our claims. In the following, we address the reviewers' specific comments (in **black**) and outline the improvements we have made to address these concerns (in **blue**). We hope that these considerations sufficiently address any remaining questions.

Reviewer #1

I thank the authors for their constructive explanation and the new CCR7 Q248I experiment. However, several of my central concerns remain only partially addressed, particularly regarding the interpretation of HA-based surface immunostaining and the extent to which the ML results can be framed as general 'receptor-intrinsic' determinants of GPCR expression rather than predictors of recombinant HA-tagged expression in HEK293T cells. I appreciate the authors' explanation that the HA tag is short and less disruptive than the larger fusion designs used in other GPCRome platforms. I also appreciate that the authors inserted the HA tag downstream of predicted signal-peptide cleavage sites for receptors with signal peptides. However, I could not find the data associated with these cleavage sites, predictions, or HA insertion constructs for the GPCRome and isoform sequences used in the study.

We apologize for this oversight and have added the Signal P predictions, the NetSurfP predictions, and the script we used to generate a consensus HA tag insertion from these outputs within a new folder entitled "signal_netsurfP_pred" in our Mendeley Data repository (https://data.mendeley.com/datasets/3b4n36z4bg/3). We have also included our final plasmid maps for each construct, which detail where the tags were inserted in each construct. We note that the plasmid maps for the final design can also be found in the folder within this directory titled "Doc S1. GPCR Plasmid Maps."

Also, the response remains largely theoretical and does not directly test whether the N-terminal HA tag alters receptor expression, maturation, or trafficking to make broad generalizable insights into GPCR biosynthesis. This is especially important in light of the inconclusive CCR7 Q248I experiment. I therefore think this concern should be addressed either experimentally or through substantially stronger qualification of the conclusions on the ML part. A focused control panel would be sufficient and need not be GPCRome-wide. For example, the authors could compare untagged and N-terminally HA-tagged versions of selected receptors expressed from the same genomic landing pad and promoter, then measure surface expression using a validated native extracellular antibody. Examples could include CCR7 as a receptor with important N-terminal determinants or CXCR1/CXCR2 as highlighted examples by the authors.

Though we have successfully used this tagging approach for several other GPCRs in the past (as previously noted), we agree that there is remaining uncertainty regarding its potential caveats. Indeed, this is a fundamental and widely appreciated caveat whenever you add an affinity tag to any protein- not just GPCRs. However, at the scale of this experiment, it is simply implausible to ascertain whether this tagging strategy works equally well for each of the 940+ receptors. Even if we were to validate this strategy for a handful of receptors, as suggested, questions would remain for the others. The reviewers noted this caveat regarding our signaling measurements in Figure S3, which showed that several of the designed receptors (with tags) were functional regardless of their expression level. The reviewers correctly noted previously that that this result could not be extrapolated to the whole panel of receptors. For this same line of reasoning, we believe a focused investigation of tagging within a limited number of receptors would not serve to address the underlying concern.

To address this concern, we noted this caveat more explicitly in the revised manuscript, as suggested. We modified the following portion of the *Discussion* section to emphasize this caveat as follows:

"*Interestingly, our machine learning models suggest the three most important topological features are the length of the N-terminus, the length of extracellular loop 2 (ECL2), and the hydrophobicity of TMD6, all of which are dynamically rewired during GPCR activation. Indeed, both the N-terminus and ECL2 mediate agonist binding, while in some niche cases ECL2 acts as an internal agonist mediating receptor signalling.[36] It is important to note that these measurements were carried out in HEK293T cells using constructs bearing a N-terminal HA tag, which could potentially perturb the biogenesis of certain receptors.*"

As it stands, the title, abstract, and discussion language still give the impression that the study reveals general sequence constraints on GPCR biosynthesis, which does not reflect the more nuanced discussion provided in the revision. I would therefore suggest that the authors consistently frame the results as identifying suggestive determinants of recombinant GPCR expression in HEK293T cells, rather than as broadly generalizable

constraints on GPCR biosynthesis. The model feature importance/ SHAP values identify features that are predictive of the measured outcome, but they do not establish that those features are causally responsible for expression differences.

The reviewer is correct. SHAP analysis cannot establish causality and it was not our intention to imply this. Furthermore, we concede that our initial claims were somewhat bold. We suspect our original conclusions are justified in light of several investigations concerning topological constraints on GPCR proteostasis published by the Schlebach lab over the past 8 years (see PMIDs: 30537820, 32181350, 34756884, 34818554, 35715957, 35850308, and 39078397, most of which were cited in this work). We now appreciate that we failed to make this point more explicitly in our original manuscript and have added the following to the Discussion section:

"*Nevertheless, the performance of these models generally recapitulates previous studies suggesting topological constraints have a dominant impact on GPCR proteostasis.*[29-31,37,38]"

Nevertheless, we agree that this is a nuanced topic and that there are likely exceptions to any generalities that emerge across an entire protein family. To address the reviewer's primary concerns, we have made the following modifications of the text in the revised manuscript. We hope this serves to address their underlying concern.

Title

Original title: "*Deep Receptor Scanning Reveals General Sequence Constraints on GPCR Biosynthesis*"

Changed to: "*Efficient Experimental Characterization of the GPCRome via Deep Receptor Scanning*"

Abstract

Original text: "*We then employ machine learning to identify specific structural features that modulate GPCR expression.*"

Changed to "*We then employ machine learning to identify specific structural features that are strongly associated with variations in GPCR expression. Our results show that many highly-expressed receptors exhibit systematic differences in hydrophobicity and secondary structure.*"

Introduction

Original text: "*Using various machine learning approaches, we comprehensively identify the general structural features that limit plasma membrane expression across the GPCR family. This experimental characterization of the GPCRome reveals generalizable insights into GPCR biosynthesis while establishing a versatile high-throughput platform that can be used to characterize numerous other aspects of GPCR activity at scale.*"

Changed to: "*Using various machine learning approaches, we comprehensively identify the general structural features that are common among highly-expressed GPCRs. This experimental characterization of the GPCRome suggests several features that potentially shape GPCR biosynthesis while establishing a versatile high-throughput platform that can be used to characterize numerous other aspects of GPCR activity at scale.*"

Results

Original text: "*Identification of Features that Limit GPCR Expression*"

Changed to: "*Identification of Molecular Features Associated with Variations in GPCR Expression*"

Original text: "*To identify key determinants of GPCR expression, we employed machine learning to survey the features associated with trends in receptor immunostaining.*"

Changed to: "*To identify molecular features associated with variations in GPCR expression, we used machine learning to evaluate trends in surface immunostaining in relation to differences in the structural features of receptors described above.*"

Original text: "*To gain insights into the structural factors that limit GPCR expression, we trained an additional series of supervised learning models to classify the expression of a subset of 452 receptors that have a complete feature set and are encoded by stable mRNA transcripts*"

Changed to: "*To focus on protein-based features associated with variations in GPCR expression, we trained an additional series of supervised learning models to classify the expression of a subset of 452 receptors that have a complete feature set and are encoded by stable mRNA transcripts*"

Original text: "*Together, our models demonstrate that combinations of these features account for most of the observed variance in GPCR expression. While also highlighting key factors that collectively give rise to tremendous variations in the abundance of mature receptors*."

Changed to: "*Together, our models demonstrate that combinations of these features account for most of the observed variance in GPCR expression while also highlighting key factors associated with variations in the abundance of mature receptors*."

Discussion

Original text: "*Finally, we curated a generalized set of descriptors for each receptor and employed machine learning to identify structural features that modulate GPCR expression*."

Changed to: "*Finally, we curated a generalized set of descriptors for each receptor and employed machine learning to identify structural features that are associated with variations in GPCR expression*."

Original text: "*The mRNA features also give rise to variations in translational efficiency, though the occupancy of the transcript does not appear to strictly dictate protein levels (Fig. 3C)*."

Changed to "*These features are also associated variations in translational efficiency, though the occupancy of the transcript does not appear to strictly dictate protein levels (Fig. 3C).*"

Original text: "*Our wider analysis of expression trends also identified a variety of structural features of the receptor proteins that modulate their plasma membrane expression (Doc. S2, Fig. 6)*"

Changed to "*Our wider analysis of expression trends also identified a variety of structural features of the receptor proteins that track with differences in their plasma membrane expression (Doc. S2, Fig. 6).*"

The new mutational experiments on CCR7 Q248I illustrate the limits of the current interpretation. The intended mutation to alter TMD6 hydrophobicity did not increase surface expression, which suggests that the importance of a feature can not as easily be extracted from the ML feature extraction, questioning the 'Reveals General Sequence Constraints on GPCR Biosynthesis' as the title claims.

We understand the reviewer's concern, but note that we did not suggest the identified features could be used to rationally improve the expression of individual receptors. Nevertheless, we have modified the title and text to more accurately reflect the nature of these observations, as noted above.

I would recommend substantially softening the ML conclusions throughout the manuscript interpreting the results as hypothesis-generating and suggestive rather than "our models demonstrate" or we have shown "generalizable insights into GPCR biosynthesis". Such claims would need to be supported more broadly and mechanistically for the reported top-ranked features in selected receptors. I also think the authors should include their CCR7 mutant experiment, to show the lack of direct translatability for inform construct engineering.

As noted above, we believe questions related to protein engineering lie beyond the scope of this investigation. Nevertheless, despite the inconclusive data for CCR7, we do believe the principles identified herein could eventually be quite useful for such an exercise. Indeed, our previous investigations into the topological energetics of both rhodopsin and GnRHR have shown that GPCR expression can, in other cases, be rationally engineered based on these simple descriptors (see PMIDs: 30537820, 32181350, 34756884, 34818554, and 35715957). Many of the identified features appear to be necessary, but not sufficient to guarantee expression on their own (see Fig. 6 D & E). For instance, hydrophobic residues in TMD6 are not alone sufficient to guarantee that a GPCR can fold and efficiently transit the secretory pathway. Receptors also need to have sufficient hydrophobicity in other helices, sufficient loop lengths for efficient translocation, and favorable networks of tertiary interactions that collectively stabilize their native fold. While the features identified herein could guide such a design effort, we suspect a robust GPCR engineering platform would likely need to generate sequences that collectively satisfy a collection of topological and structural constraints. As this lies far beyond the scope of this manuscript, we do not believe it makes sense to further expand our discussion of the findings.

Its actually also unclear to me how the CCR3 glycosylation and CXCR1 ER-retention motif experiments relate to the ML analysis presented in Figure 6. The tested features (N-terminal glycosylation) are not among the SHAP-ranked predictors and hence to not support the claims made from the ML model, rather the opposite.

We agree. We expanded the scope of these supplemental measurements to explore whether it may be worth incorporating descriptors related to the presence of certain motifs into our ML models. We ultimately decided this would not add to the manuscript in a meaningful way. We shared these results for the sake of transparency, but apologize for any confusion this may have caused.

My remaining concern is not with the utility of the resource, which I find to be a valuable platform, but with the scope and validity of the biological claims.

We thank the reviewer for their perspectives and for helping us to identify the appropriate scope of our claims.

Reviewer #1 (Remarks on code availability):

Already discussed during the first review.

We thank the reviewer for taking the time to carefully review our code.

Reviewer #2

The authors have sufficiently addressed my comments to the first version of the manuscript.

We thank the reviewer for carefully considering both drafts of the manuscript.

Reviewer #3

While my comments have mostly been addressed, I remain skeptical about the value of the proposed approach. Even if suggested GPCR modifications could improve biosynthesis, they might not be compatible with functionality. The newly included application data already indicate clear limitations in the reliability of obtained predictions. Numerous related AI-based concepts are currently coming up but they unfortunately often lack support by a sufficient record of experimental data. In principle, the authors propose a prediction process but leave it up to the reader to prove its usefulness.

We thank the reviewer for their perspectives, but again humbly disagree with their assessment of our intent. This work was not intended to deliver a platform to enhance GPCR biosynthesis and/ or function, and we in no way implied such in either draft of the manuscript. We also note that we did not train these ML models to maximize their predictive power (note the limited feature sets), but rather to identify and interpret trends within a relatively large amount of unique data. Had we not included this analysis, we believe this paper would simply constitute the description of a new method and a corresponding data dump. Instead, we believe the application of these relatively simple models provides the community with a versatile new companion diagnostic tool that can be used to interpret any future outputs of this assay. Others are certainly welcome to incorporate our findings into more sophisticated GPCR engineering platforms, if that is of interest.
